# Localisation of RNAs into the Germ Plasm of Vitellogenic *Xenopus* Oocytes

**DOI:** 10.1371/journal.pone.0061847

**Published:** 2013-04-23

**Authors:** Sarbjit Nijjar, Hugh R. Woodland

**Affiliations:** School of Life Sciences, University of Warwick, Coventry, United Kingdom; Radboud University Nijmegen, The Netherlands

## Abstract

We have studied the localisation of mRNAs in full-grown *Xenopus laevis* oocytes by injecting fluorescent RNAs, followed by confocal microscopy of the oocyte cortex. Concentrating on RNA encoding the *Xenopus* Nanos homologue, *nanos1* (formerly *Xcat2*), we find that it consistently localised into aggregated germ plasm ribonucleoprotein (RNP) particles, independently of cytoskeletal integrity. This implies that a diffusion/entrapment-mediated mechanism is active, as previously reported for previtellogenic oocytes. Sometimes this was accompanied by localisation into scattered particles of the “late”, *Vg1*/*VegT* pathway; occasionally only late pathway localisation was seen. The *Xpat* RNA behaved in an identical fashion and for neither RNA was the localisation changed by any culture conditions tested. The identity of the labelled RNP aggregates as definitive germ plasm was confirmed by their inclusion of abundant mitochondria and co-localisation with the germ plasm protein Hermes. Further, the *nanos1*/Hermes RNP particles are interspersed with those containing the germ plasm protein Xpat. These aggregates may be followed into the germ plasm of unfertilized eggs, but with a notable reduction in its quantity, both in terms of injected molecules and endogenous structures. Our results conflict with previous reports that there is no RNA localisation in large oocytes, and that during mid-oogenesis even germ plasm RNAs localise exclusively by the late pathway. We find that in mid oogenesis *nanos1* RNA also localises to germ plasm but also by the late pathway. Late pathway RNAs, *Vg1* and *VegT*, also may localise into germ plasm. Our results support the view that mechanistically the two modes of localisation are extremely similar, and that in an injection experiment RNAs might utilise either pathway, the distinction in fates being very subtle and subject to variation. We discuss these results in relation to their biological significance and the results of others.

## Introduction

The oocytes of Metazoa are usually highly polarised cells in which mRNAs are localised and stored as part of a mechanism to enable rapid early diversification of embryonic cells. Since the mechanisms of polarisation are not unique to oogenesis, but later operate in somatic cells, the tractability of embryonic systems has made the oocytes of many model species paradigms for the study of general polarisation and RNA localization mechanisms [1], [2]. Although many advances in our understanding of oocyte polarisation have relied on the genetic methods applicable to *Drosophila* and *Caenorhabditis elegans*, the *Xenopus* oocyte has also proved a productive model for studying RNA localisation because the cells are large and readily cultured in vitro. Tagged or labelled RNA molecules may readily be injected into the cell and their localisation followed to positions that mirror endogenous molecules [3]–[7].

Endogenously expressed RNAs may be localised either to the animal or vegetal pole of *Xenopus* oocytes; localisation to the animal pole is relatively weak and is non-cortical, whereas the concentration of vegetal RNAs is much stronger and these RNAs are anchored in the cortex [3]. In the vegetal hemisphere of the full-grown oocyte two distinct locations for stored RNAs have been described: **(1)** The germ plasm, a field of structures consisting of tightly bound cortical aggregates of RNPs and other components. It directs the development of the future germline. Classically germ plasm has been identified with cytological stains or by in situ hybridisation using chromogenic detection methods. These approaches identify large structures that have been called germinal granules. Electron microscopy shows that these consist of various components, including RNPs, mitochondria and cytoskeleton [8]. In ultrastructural analyses these RNPs tend to be called granules, whereas at low resolution the term is applied to the aggregates as a whole, e.g. Heasman, Quarmby and Wylie [9]. The latter authors also called the oocyte aggregates “islands”, as did Kloc et al. [10]. Because there is potential confusion in using the word “granules”, we describe the small RNA-containing structures resolved by the confocal microscope as particles and the aggregates as germ plasm islands. **(2)** The *Vg1*/*VegT* compartment, widely scattered RNP particles that are dependent on at least two RNAs, *VegT* and *Xlsirts*, for their structure [10]–[12]. This class of RNAs is involved in endoderm and mesoderm specification and embryonic axis patterning.

The existence of these two compartments has been established by using in situ hybridisation to analyze the distribution of endogenously stored RNAs, and complemented by conducting RNA localisation experiments with injected tagged or fluorescently labelled RNAs, often testing the localisation ability of regions within the 3′UTR. Many endogenous germ plasm RNAs are initially localised into the single large Balbiani body, or mitochondrial cloud, of early previtellogenic oocytes. The Balbiani body is a dense structure composed of messenger RNPs, cytoskeleton, endoplasmic reticulum and mitochondria. While it has been identified in only a proportion of animals, this number is steadily growing and it may be universal. For example it was recently described in *Drosophila* and mouse oocytes, even though the latter do not form germ plasm [13], [14]. Therefore an element of caution is required when equating the Balbiani body with germ plasm, indeed *Wnt11* RNA initially localises to the Balbiani body, but subsequently it is present in late pathway granules; in contrast other RNAs only localise to the Balbiani body in late previtellogenesis (*Hermes, Fatvg, Grip2*) [11], [15]–[18]. In stage II previtellogenic oocytes the Balbiani body moves to the vegetal cortex of the oocyte, forming a field of cortical granular islands now called germ plasm. Thus in *Xenopus* the germ plasm is continuous with the Balbiani body, even if its composition changes. While RNA localisation (at least of injected RNAs) into the Balbiani body is mediated by diffusion/entrapment [19], [20], like that of *nanos* mRNA to germ plasm in *Drosophila* [21], other components of the cloud use microtubules [22].

Initially RNAs localised to the *Vg1* compartment RNAs are generally distributed throughout the cytoplasm, but are excluded from the Balbiani body. At the onset of yolk accumulation (vitellogenesis), they become localised into particles dispersed throughout the vegetal cortex; this form of localisation was therefore called the “late pathway” [23], [24]. It was shown that injected late RNAs, initially *Vg1*, localise by this pathway in mid stage oocytes, using microtubule transport [24]–[26]. It is sometimes said to require vitellogenin endocytosis, but injected RNAs will localise so long as serum is present [26]. It was originally reported that the late pathway was not active in full-grown oocytes [26], so experiments on this pathway have usually been conducted with mid-oogenesis oocytes (stage III–IV), but it now seems that this pathway may be active even in late oogenesis [27].

Recent data suggest that the proposed dichotomy into two pathways is an over-simplification. As already mentioned, some RNAs localise to the Balbiani body only in late pre-vitellogenesis (*Hermes, Fatvg, Grip2*), while others localise both to the Balbiani body and throughout the oocyte, then later are found in the germ plasm (*Germes*). *Wnt11* RNA initially localises by the early pathway, but subsequently it is present in late pathway particles. Lastly, the 3′UTRs of early RNAs have been reported to localise like late ones when injected into vitellogenic oocytes [24], [28], [29] and conversely late RNA UTRs may earlier direct localisation to the Balbiani body [30]. In many of these studies (including our own) relatively low resolution imaging techniques were used, so a re-examination of RNA localisation at later stages seems timely.

Localisation to the Balbiani body by the early pathway has not been studied as intensely as the late pathway. The fact that in later oocytes early RNAs are said to localise by the late pathway suggests a mechanistic overlap between the two pathways, and they do require very similar sequence motifs. However, at least in reported microinjection experiments, the late pathway involves transport on microtubules, whereas the earlier one occurs by diffusion/entrapment [20]. *XNIF* RNA illustrates the overlap between the pathways: an element in its 5′UTR brings about localisation to the vegetal cortex at stage III, but not to the mitochondrial cloud at stage I, unless the UTR element is duplicated [31], suggesting that critical numbers of repeats may differentiate the pathways. One also has to bear in mind that the Balbiani body is not identical to later germ plasm; in particular, as already mentioned, RNAs that are localised into one structure are not necessarily localised into the other, or they change their localisation vis a vis the Balbiani body as time passes. Moreover, distinct 3′UTR elements in *nanos1* mRNA have been identified that locate the RNA to the Balbiani body [32] and to particles within it [33]. This gives good reasons to find ways to study late stages, when the RNA compartments are distinct. The hypothesis of temporally distinct mechanisms suggests that the two pathways cannot be studied simultaneously in the same cell, and this may have contributed to the lack of clarity about the molecular nature of the two localisation mechanisms.

In *Xenopus,* oogenesis lasts for months and large oocytes may be maintained for years in the absence of ovulation. It seems intrinsically unlikely that localisation would occur so early during oogenesis without any further activity, since biological structures usually need continual maintenance. We have previously found that several proteins will localise into germ plasm at late stages, specifically Germes [34] and xPix1, now referred to as Poc1B [35]. For these reasons we have reinvestigated the localisation of vegetal mRNAs and the RNA-binding protein Hermes into germ plasm.

We have studied the localisation of several germ plasm RNAs injected into mid-stage and full-grown *Xenopus* oocytes. Because it has been intensively studied by others we have particularly focused on *nanos1* mRNA (formerly known as *Xcat2*). The *Xenopus* protein Nanos1 is a homologue of Nanos, a protein encoded by a widely distributed germline RNA, first identified in *Drosophila* [36]. It functions as a translational repressor and is essential for germline development in *Xenopus*, acting as a translational repressor [37], [38]. The mRNA encoding Nanos1, when labelled with Cy5-UTP or digoxigenin, localises to the Balbiani body of previtellogenic oocytes by diffusion and entrapment [19], [20]. We have also expanded our study to include *Xpat* and *Xvelo1* RNAs. Xpat is a germ plasm RNA whose protein is also present in germ plasm, in particles which we show here are distinct from those containing Hermes protein and the various germ plasm RNAs tested, including that encoding Xpat itself [22], [29]. Machado et al. were able to demonstrate that Xpat protein is capable of generating fields of aggregated particle resembling germ plasm [22]. *Xvelo1*, is a vegetally localized RNA whose function is unknown, but it encodes the homologue of Bucky ball, which is essential for germ plasm formation and oocyte polarity in zebrafish [39], [40].

In this study we find that germ plasm RNAs usually localise into the RNP particles of germ plasm islands in large oocytes. Here they are co-localised with the RNA-binding protein Hermes, a known germ plasm component [41], [42]. Interestingly we found that several “late” pathway RNAs may also enter germ plasm structures. Rather than many kinds of RNA being localised by the late pathway in advanced oocytes, we find that, while this may also occur, late RNAs frequently mimic germ plasm RNAs by entering germ plasm by a cytoskeleton-independent mechanism.

## Results

### Localisation of Injected Xenopus Nanos1 and Xpat mRNAs into the Germ Plasm of Stage V/VI, Large and Full-grown Oocytes

**Figure 1:**
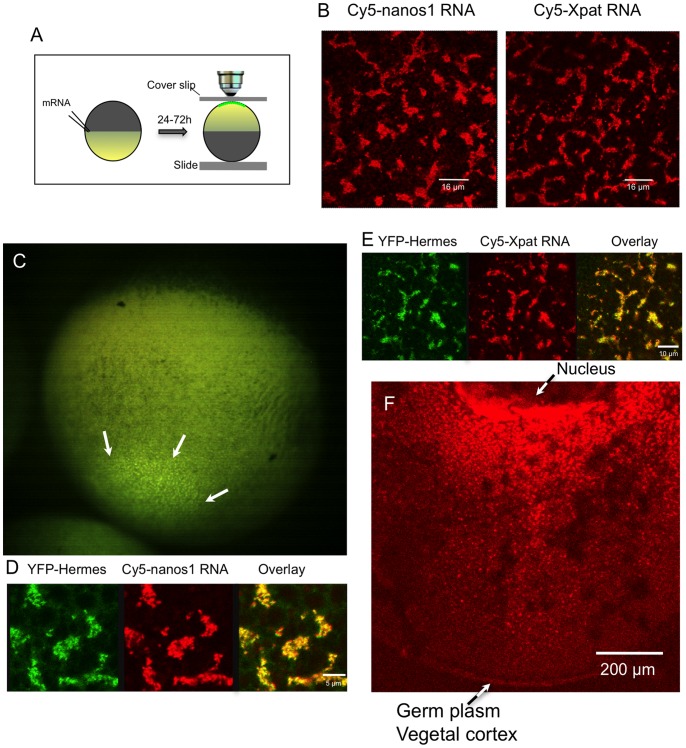
Localisation of ‘early pathway’ mRNAs and Hermes protein into the germ plasm of stage VI oocytes. A. The procedure for examining the cortical distribution of labelled RNAs. After injection and incubation of oocytes in OCM (with or without vitellogenin-containing serum) for 24 to 72 h, oocytes were held in an inverted position between a slide and a coverslip, in a chamber made with a latex spacer. They were then examined by confocal microscopy using a 40× oil-immersion lens. B. Full-length Cy5-labelled *nanos1* and *Xpat* RNAs localise in islands of particles at the vegetal pole 48 h after injection. C. Low power stereo microscope view from the side of the vegetal pole of a whole stage VI oocyte, 24 h after injection with mRNA encoding YFP-Hermes. YFP-Hermes protein is clearly localised to a field of fluorescent islands at the vegetal pole (white arrows), typical of germ plasm markers. In the stereo microscope the depth of focus is large so that a much larger area than that occupied by the germ plasm is in focus. D,E. In these islands Cy5-labelled *nanos-1* and *Xpat* RNA’s co-localise with YFP-Hermes protein, following co-injection of mRNA encoding YFP-Hermes. All the above oocytes were visualized live, as they are in later figures unless otherwise stated. F. Internal distribution of Cy5-*nanos1* RNA between the nucleus and the vegetal cortex of a fixed stage VI oocyte. Following injection of RNA, oocytes were cultured for 48 h in OCM, fixed and hemisected with a scalpel prior to visualization by confocal microscopy using a Z-stack.

We initially investigated the localisation of Cy5-labelled *nanos1* RNA injected into stage V–VI oocytes. In the experiments described here, unless otherwise stated, oocytes were maintained within intact follicles, including both follicle and surrounding thecal cells, to simulate natural conditions as closely as possible Oocytes were cultured in the same OCM medium originally used by Yisraeli and Melton [23], except that the yolk protein precursor vitellogenin was not routinely included. They were then usually examined alive by confocal microscopy, as shown in Figure 1A.

Three distinct modes of localisation were evident in injected oocytes (Table 1); the first was exclusively into germ plasm islands (Pattern I, Figure 1B). In the second, germ plasm localisation was accompanied by variable numbers of scattered particles containing Cy5-*nanos1*, but not YFP-Hermes (see below) (Pattern II). An example is shown later in Figure Four. Occasionally, we observed an exclusively late pathway mode of localisation (Pattern III). The frequencies of these patterns is summarised in Table 1. Typically, in experiments with oocytes derived from a given female a single outcome was usually seen. Similar results were seen when the *nanos1* 3′ UTR alone was injected, except that the proportions showing Patterns II and III were higher.

**Table 1:** Summary of localisation patterns displayed by injected RNA.

	Number and proportion of oocytes displaying indicated localisation pattern.			
Injected RNA and oocyte stage.	Pattern I (Germplasm only)	Pattern II (Germ plasm+late pathway)	Pattern III (latepathway only)	Negative
*nanos1 FL* Stage VI (N = 53)	268/332 (81%)	39/332 (12%)	9/332 (2.7%)	16/332 (5%)
*nanos1 3′ UTR* Stage VI (N = 30)	132/243 (54%)	31/243 (13%)	50/243 (21%)	30/243 (12%)
*nanos1 FL* Stage IV (N = 8)	34/63 (54%)	17/63 (27%)	12/63 (19%)	0/63 (0%)
*nanos1 3′ UTR* Stage IV (N = 7)	9/56 (16%)	4/56 (7%)	32/56 (57%)	11/56 (20%)
*Xpat FL* alone, Stage VI (N = 9)	25/49 (51%)	15/49 (31%)	0/49 (0%)	9/49 (18%)
*Xpat 3′ UTR* Stage VI (N = 7)	30/43 (58%)	3/43 (4%)	5/43 (0%)	5/43 (17%)
*VegT* Stage VI (N = 2)	7/10 (70%)	0/10 (0%)	0/10 (0%)	3/0 (30%)
*Vg1* Stage VI (N = 10)	42/82 (51%)	11/82 (13%)	5/82 (6%)	24/82 (29%)
*Xvelo1* Stage VI (N = 2)	4/6 (67%)	2/6 (33%)	0/6 (0%)	0/6 (0%)
YFP-Hermes alone, Stage VI (N = 18)	72/73 (99%)	1/73 (1%)	0/73 (0%)	0/73 (0%)
YFP-Hermes alone, Stage IV (N = 3)	15/15 (100)%	0/15 (0%)	0/15 (0%)	0/15 (0%)

In each table cell the number of oocytes showing a given localisation with respect to the total number of injected oocytes is shown. N = the number of experiments using oocytes from different females. Cy5 or Cy3-labelled RNAs were routinely injected, with or without RNA encoding YFP-Hermes. The YFP-Hermes alone denotes localisation by the translated protein following injection of the RNA. FL; full length.

It is important to emphasise however that not all injected RNAs were localised. We show later in Figure seven that mutants of *nanos1* RNA, specifically containing the ORF, and *nanos1-3′Δ4*, fail to show any localisation at all. These sequences were cloned in the vector pSPJC2L [43] within short stabilising UTR sequences. In separate experiments we showed that Cy5- labelled RNA transcribed from a similarly cloned *GFP-Xpat*-ORF fusion, failed to localise (data not shown). Separately we showed that mRNA from this construct translates into large amounts of protein, so it must be stable [22].

Routinely we injected 3 ng of Cy5-labelled RNA into each stage VI oocyte. In proportion to volume we injected about the same amount as did Messitt et al. into stage III oocytes [44], since stage VI oocytes are 50× larger by volume than those at stage III. In order to discount any effects on localisation by the amount of injected RNA we injected four times less than this and found that localisation was identical. At ten times less RNA 2/6 oocytes showed localisation similar to that seen with 3 ng RNA, the others being negative. So we have no evidence that we affect localisation by saturating the system.

It was important to establish that the aggregated particulate structures were indeed true germ plasm. Hermes, an RNA-binding protein containing a single RNA recognition motif (RRM) [45], is a known protein constituent of *Xenopus* and zebrafish germ plasm particles [41], [46]. In addition to the Balbiani body, *Hermes* RNA is localised in germ plasm particles in the vegetal cortex of vitellogenic oocytes [41], [42]. Furthermore, endogenous *nanos1* mRNA has been detected as a component of immunoprecipitated Hermes RNP complexes from early stage oocytes [42]. We made constructs of Hermes in which the coding region was fused to YFP, either at the N or C terminus. When mRNA encoding these was injected into stage V or VI *Xenopus* oocytes both fusion proteins were expressed at high levels and they were localised to the vegetal cortex in a field of islands typical of germ plasm. The depth of focus in the low power image shown in Figure 1C (captured using a stereo microscope) is sufficient to show clearly that the field is restricted to the centre of the vegetal pole. When this field was examined by confocal microscopy, YFP-Hermes was clearly present in granular islands, and co-localised with injected Cy5-*nanos1* mRNA (Figure 1D). Thus Figure 1C would also represent how Cy5*-nanos1* would appear if the latter were visible in the stereo microscope. Table 1 shows that the Hermes fusions localised exclusively to germ plasm particles in the cortex of the oocyte. These observations mirror the patterns seen for endogenously expressed Hermes protein and *nanos1* RNA [41], and we show later that the co-localisation holds for endogenous Hermes and injected Cy5-*nanos1* RNA in the vegetal cortex as studied by the methods used here. It is notable that there are no YFP-Hermes fluorescent particles in the cortex that do not contain Cy5-*nanos1*.

The ability of YFP-Hermes fusion proteins to behave in a manner similar to the endogenous protein was confirmed by staining injected oocytes with Hermes anti-serum and an Alexa 633 secondary antibody. The fluorescent signals corresponding to endogenous and exogenous protein were coincident (not shown), indicating that YFP-Hermes is incorporated into all of the endogenous Hermes particles. Since Cy5-*nanos1* RNA was coincident with YFP-Hermes this RNA must also have been incorporated into existing RNPs, within existing germ plasm islands.

In this report, when it is stated that molecules were co-localised within specific particles, we are obviously limited by the resolution of the light microscope. However the particles were large enough to be resolved (∼1 µm), the fluorescent colour coincidence was homogeneous and when the particles were observed to move around by Brownian motion or perhaps microtubule transport, they behaved as unitary structures. There is, therefore no reason to believe that the particles were not single RNPs, like those seen in electron microscope studies, containing both Cy5-*nanos1* and YFP-Hermes protein.

**Figure 2:**
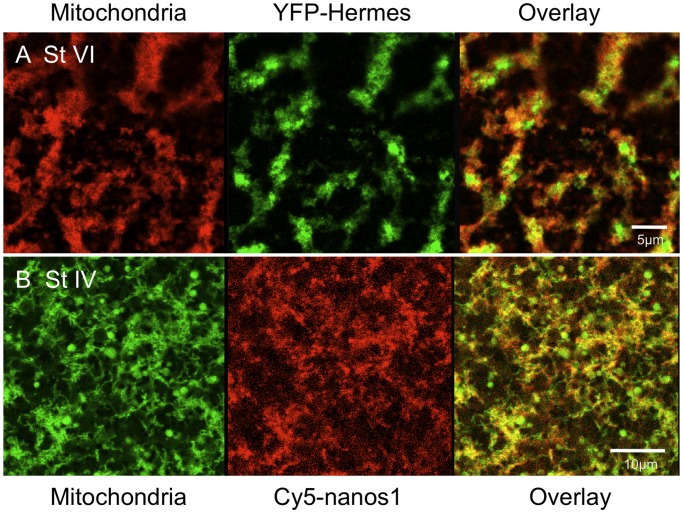
YFP-Hermes localises into particles in islands containing concentrated mitochondria. A. Stage VI oocytes were injected with RNA encoding YFP-Hermes and after 18 h mitochondria were stained with TMRE [22], prior to visualisation of the vegetal cortex, as in Figure 1A. B Stage IV oocytes were injected with Cy5-*nanos1* RNA and after 24 h the oocytes were stained with TMRE and analysed as in A.

A diagnostic feature of germ plasm in diverse species is that it contains dense mitochondrial aggregates. We therefore stained oocytes with the dye TMRE, which is sequestered by mitochondria. Figure 2A shows that in live oocytes YFP-Hermes protein was localised into particles within the same islands enriched in mitochondria, although the two YFP and TMRE were not exactly coincident, as would be expected. This confirms the identity of the fluorescent islands (sometimes called germ granules) as germ plasm. Since both YFP-Hermes and Cy5-*nanos1* exactly co-localise in aggregated particles, they must also both be associated with the mitochondrial aggregates and hence must be localised in germ plasm islands. Other experiments confirmed that the Cy5-*nanos1* co-localised with endogenous Hermes, stained with antiserum (data not shown), and we show later that Xpat, another germ plasm protein was concentrated in these islands. We have found that *nanos1* RNA localised in the same way whether labelled separately with Cy5-UTP, Cy3-UTP or Aminoallyl-UTP-ATTO390.

Previously early pathway RNAs had been reported to follow the late pathway when injected into vitellogenic oocytes. In the experiments just described there were some differences in procedures adopted by us and those usually followed by others. We have routinely used OCM lacking high concentrations of vitellogenin within *Xenopus* serum, although other workers often add this. Serum containing vitellogenin was originally reported to be important for *Vg1* RNA localisation [23], although these authors later reported that it was only serum itself that was essential [26]. It is also usual to use oocytes freed from their follicles using collagenase, although this was not done in the original experiments of Yisraeli and Melton [23], [26]. We tested whether adding vitellogenin-containing serum or removing follicle cells (either manually or with collagenase) affected the localisation of *nanos1* RNA and/or Hermes protein. There was no noticeable effect of changing any of these conditions, either individually or in combination, in any given experiment.

We tested if the observations just described extended to another germ plasm RNA. Previous data showed that endogenous *Xpat* RNA was localised in the germ plasm of full-grown oocytes, just like its protein [8], [22], [29]. Furthermore, when it was microinjected, lac-tagged *Xpat* RNA or its 3′UTR alone, localised to the vegetal cortex of mid-stage oocytes [29]. Here we find that injected Cy5-*Xpat* RNA was localised to the germ plasm of stage VI oocytes, just like *nanos1* RNA (Fig. 1E).

Although we focused on localisation into the oocyte vegetal cortex, we also observed that Cy5-*nanos1* RNA was localised into particles in the internal oocyte cytoplasm. Figure 1F shows an accumulation of RNA on the vegetal side of the oocyte nucleus and in particles in the oocyte cytoplasm extending all the way down to the vegetal cortex. This is reminiscent of a previous report on stage III oocytes, which described streams of Cy5-labelled *Vg1* RNA localisation elements between the nucleus and oocyte cortex [44]. Similar structures were seen upon detection of injected lac RNA-tagged *Xpat* UTR by conventional in situ hybridisation [29].

### Intact Microtubules and Microfilaments are not Needed for Localisation of Germ Plasm RNAs in Stage V/VI Oocytes

**Figure 3:**
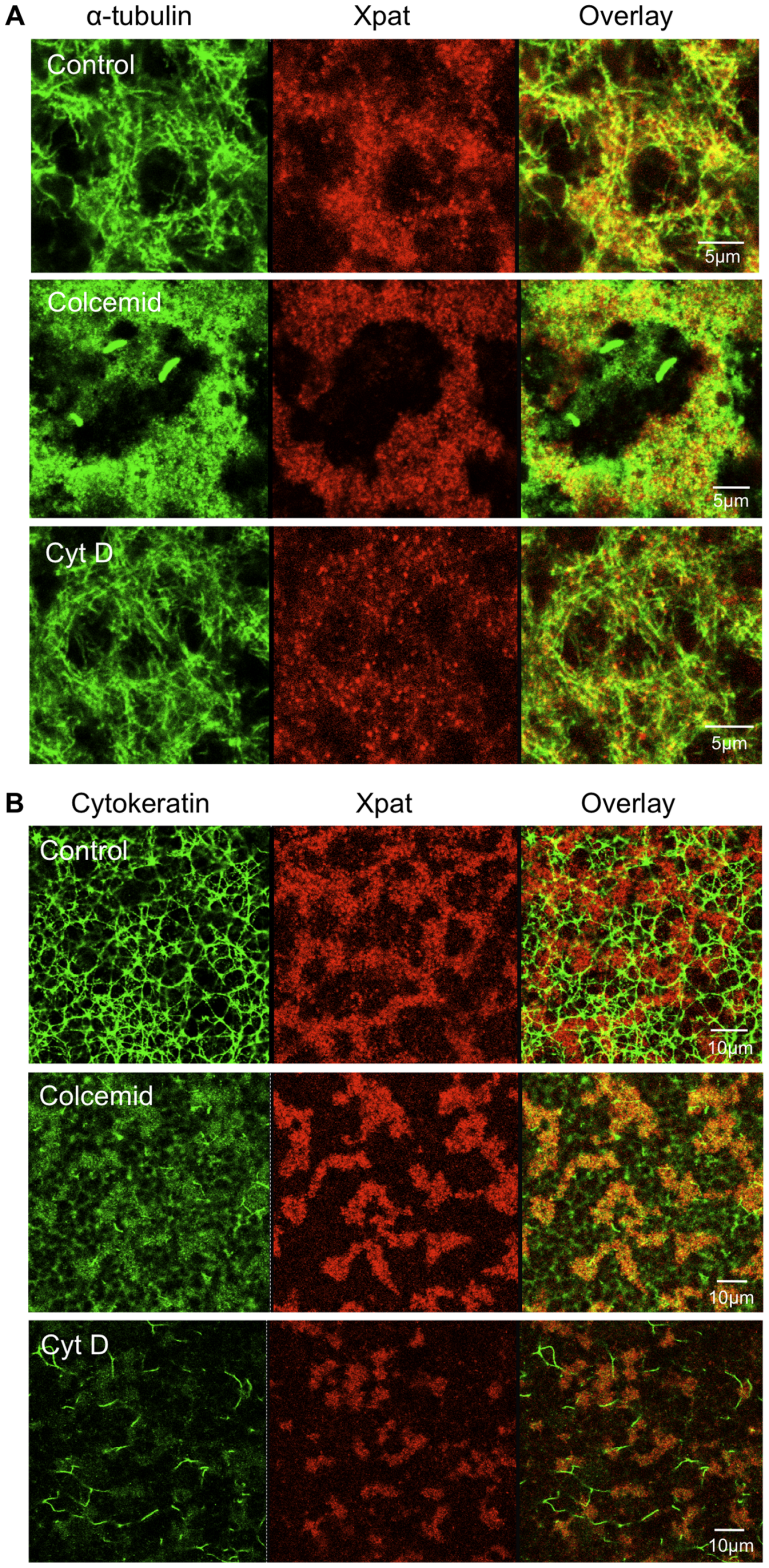
Disruption of micro- and intermediate filaments and its effect on germ plasm structure. Oocytes were cultured for 24 h with the inhibitors shown. They were fixed and Xpat protein particles were stained with purified antibodies [22]. Following Xpat staining oocytes were co-stained for microtubules, A, or cytokeratins, B.

It has been reported that localisation of *nanos1* RNA to the Balbiani body of stage I oocytes does not need an intact cytoskeleton [19], [20]. We added disruptive agents (colcemid or nocadazole for microtubules; cytochalasin D for microfilaments; see Methods) to test if this was true of localisation to the germ plasm at late stages. Figure 3A shows that after 24 h microtubules were almost entirely removed with colcemid, and nocadazole treatment gave similar results (not shown). While there was staining with the tubulin antibody, it was not filamentous; unpolymerised tubulin was concentrated around the germ plasm because the surrounding cytoplasm is packed with yolk granules, which exclude other components. The disruption of microfilaments by cytochalasin D had little effect on microtubule integrity. However, the staining of oocytes with antibody C11 revealed that cytokeratin intermediate filaments were removed by colcemid treatment and cytochalasin D had a similar effect (Figure 3B).

**Figure 4:**
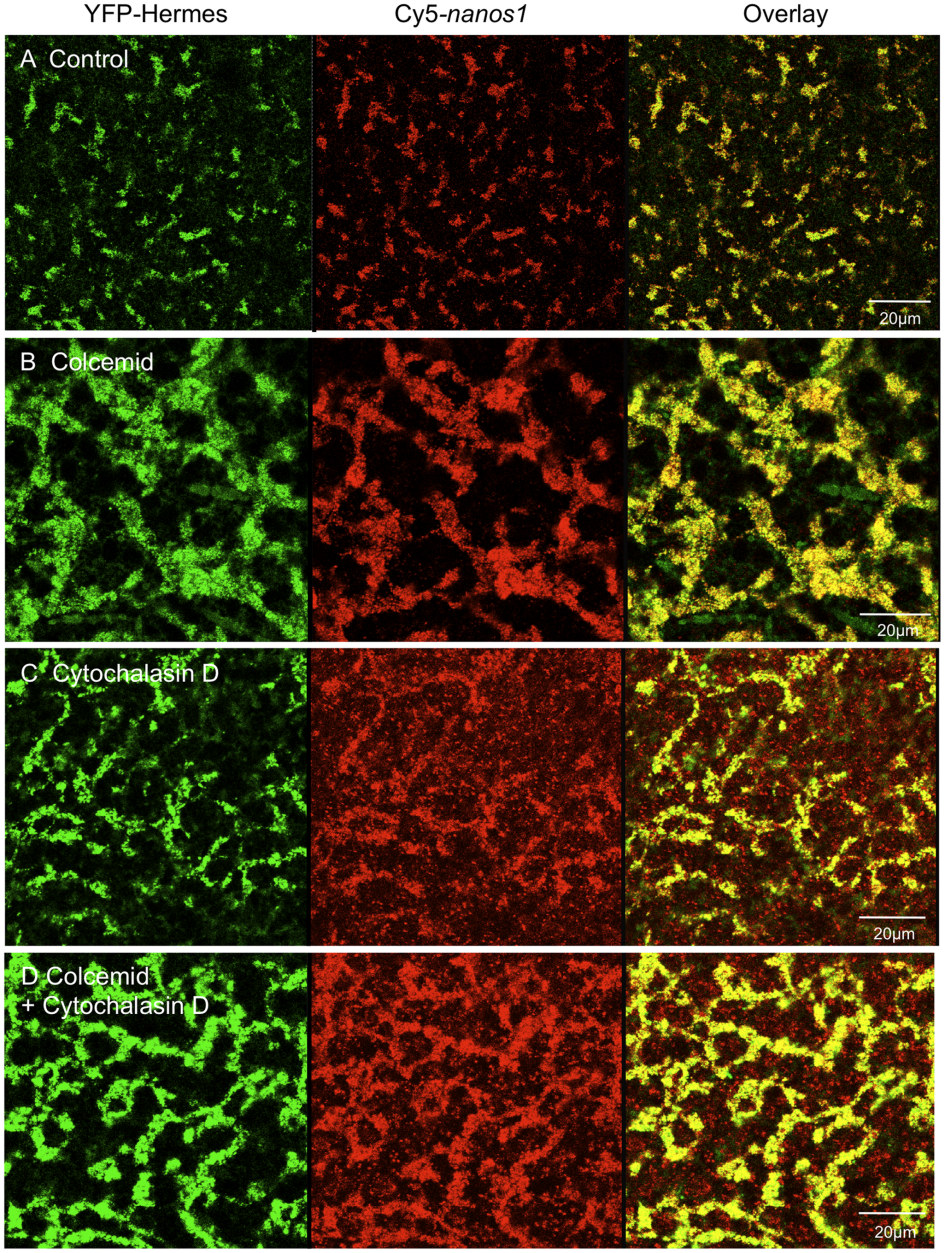
Disruption of the tubulin and actin cytoskeletons does not prevent localisation to the germ plasm. Oocytes injected with Cy5-*nanos1* and *YFP-Hermes* RNAs were treated with colcemid, cytochalasin D or both. After 48 h the vegetal cortex was examined by confocal microscopy. A. Control oocytes. Oocytes were treated with: B. Colcemid. C. Cytochalasin. D. Both inhibitors. (Nocodazole produced results similar to B, not shown).

In Figure 4 the localisation of injected Cy5-*nanos1* RNA and YFP-Hermes protein is seen to proceed efficiently in the presence of the colcemid and cytochalasin D concentrations used above (nocadazole produced results similar to colcemid). Thus intact microtubules and microfilaments are not necessary for localisation to vegetal RNP particles, at least within the experimental time scale required for injected *nanos1* RNA localisation (clearly on a longer time scale the whole polarity of the oocyte depends on the cytoskeleton). The existence of microtubules resistant to disruption by agents like colcemid has been suggested, however the tubulin staining in Figure 3A shows that there are so few remaining in the germ plasm region that microtubules cannot be essential for the localisation studied here. Furthermore Figures 3 and 4 also show that cytokeratin filaments are not required for the localisation of *nanos1* RNA and YFP-Hermes protein to germ plasm, since colcemid and cytochalasin D incidentally disrupted these. It will be noticed that colcemid caused the germ plasm islands to run together into a reticular structure. In these oocytes there were a few scattered Cy5-*nanos1* particles lacking Hermes, as will be seen below in some stage IV oocytes (Pattern II). It is likely that these were late pathway particles and their frequency was greatly increased by cytochalasin D treatment. While the cytochalasin effect was not observed in experiments in which no late pathway granules were labelled in controls untreated with cytochalasin D, the possibility that actin filaments have a role in discriminating the two pathways might be worth investigating further.

**Figure 5:**
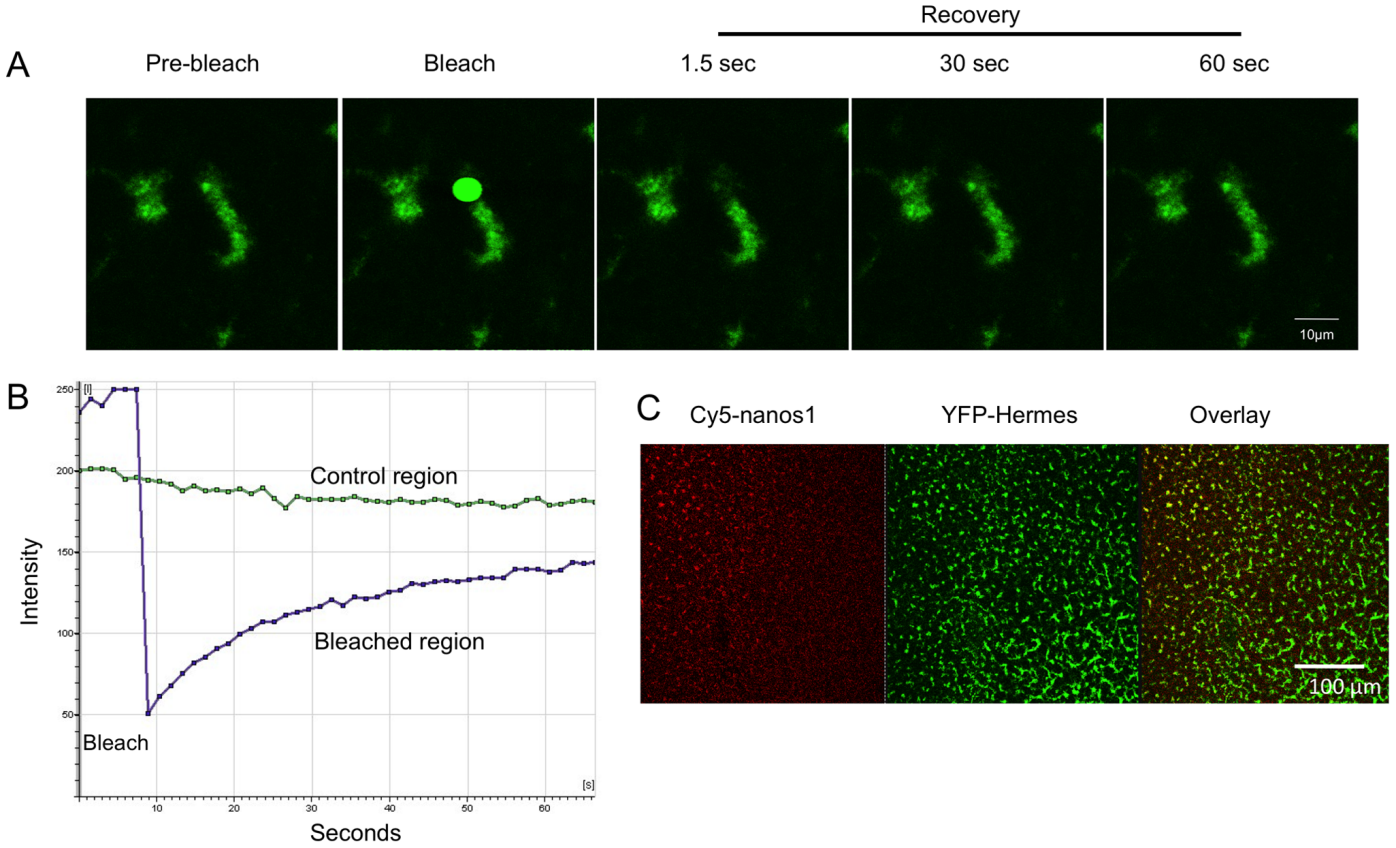
FRAP experiment showing that Hermes protein in germ plasm exchanges with a cytoplasmic pool YFP-Hermes. A. Confocal images, before during and after the bleaching step. B. Quantification of the bleached area and a small unbleached control region. C. Low power image to show that injected RNA diffuses more slowly than protein. Oocytes were co-injected at the equator with Cy5-*nanos1* and *YFP-Hermes* RNAs (The latter does not have its own UTRs, so would not localise). After 48 h the vegetal pole was visualised and it is seen that the Cy5-*nanos1* RNA has diffused more slowly from the injection point, at the top left, than Hermes protein.

If cytoskeletal transport is not essential for the localisation of injected RNAs and newly expressed Hermes protein, it suggests that diffusion and exchange into pre-existing particles occurs. We have tested this by FRAP analysis of YFP-Hermes. Thus Figure 5A,B shows that when we bleached a small area of YFP-labelled particles in germ plasm the fluorescence substantially recovered in less than a minute. This implies that there is continuous exchange between Hermes in the particles and a pool of fluorescently labelled protein in the surrounding cytoplasm. Similar experiments with Cy5-*nanos1* failed to show recovery, indicating that this RNA was more stably incorporated into germ plasm. Given that this RNA seems to localise by diffusion, either the RNA exchanges much more slowly than Hermes protein throughout the cytoplasm, or the germ plasm is the final target of this RNA and therefore there is little exchange only within cortical particles. Perhaps a mixture of the two possibilities is most likely.

Previously it has been reported that RNAs diffuse more slowly than proteins in oocytes [47]. Thus, if the injected RNAs and the Hermes protein diffuse independently one would expect Hermes, a relatively small protein, to spread faster through the oocyte than Cy5-*nanos1* RNA. We were able to confirm that this is indeed the case, as exemplified by the low power image shown in Figure 5C. After co-injecting Cy5-*nanos1* RNA and RNA encoding YFP-Hermes into the equator a large area of the vegetal pole was imaged. The YFP-Hermes is evenly distributed over the whole vegetal pole, whereas the Cy5-*nanos1* RNA is concentrated over only part. This result is consistent with the idea that *nanos1* RNA and Hermes protein spread by diffusing separately. The slower movement of Cy5-*nanos1* could also relate to its more stable incorporation into germ plasm. It is also possible that most of the diffusion of Cy5-RNA is through internal cytoplasm and the RNPs might be less stable there, but we cannot image this in FRAP experiments (see Discussion). [Of course the situation is complicated in that one is comparing an injected RNA with a protein translated from a co-injected RNA. The *YFP-Hermes* RNA lacked its own UTRs, so would not itself have been localised. The restriction in the ability of the *Hermes* mRNA to diffuse would, if anything, be expected to reduce the area over which the Hermes protein diffused].

### Localisation in Stage IV Oocytes

Another notable difference between the experiments described so far and those of other workers is that they were performed with large oocytes (stage V–VI), whereas others use early to mid-vitellogenic oocytes (stage III–IV). This is probably a consequence of the original report that *Vg1* mRNA was not localised to the vegetal cortex in full-grown oocytes [23].

**Figure 6:**
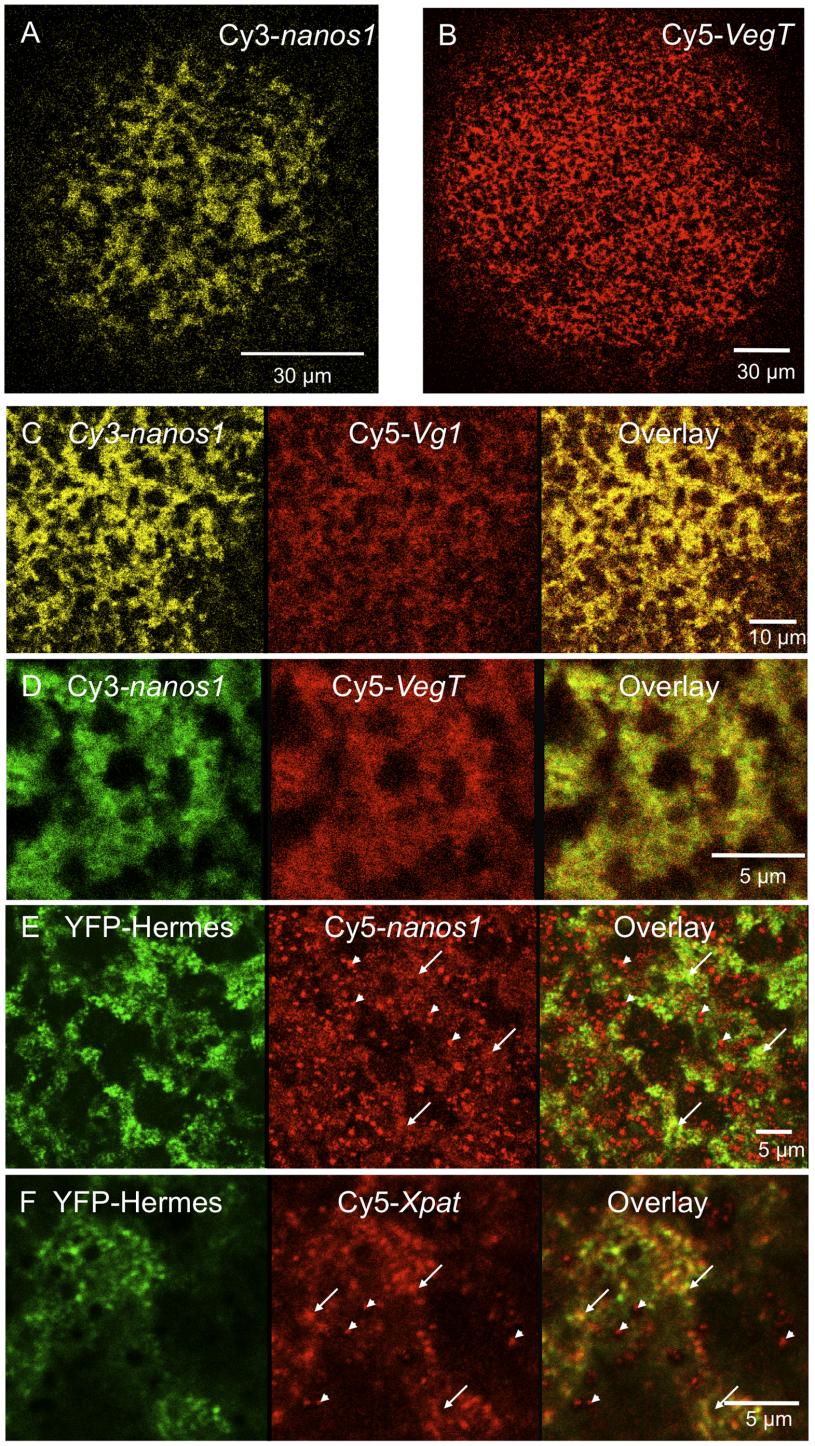
Localisation of RNAs in stage IV oocytes. The indicated RNAs were injected into stage IV oocytes and cultured in the presence of vitellogenin for 48 h. A–D show pattern I, with exclusively germ plasm localisation. A field of Cy3-*nanos1* (A) and Cy5-*VegT* RNA (B) aggregates was seen at the vegetal pole at low magnification. C and D show higher power views of these oocytes. E and F show higher power views of oocytes showing Pattern II, a mixture of germ plasm localisation (RNP particles containing Hermes) and late pathway localisation (particles lacking Hermes). To illustrate our identification of structures formed by the activity of the two pathways of RNA localisation, in panels E and F examples of germ plasm are identified with arrows and late pathway particles with arrowheads. The former show coincident Cy5 and YFP signals, but the latter lack the YFP-Hermes.

We therefore tested the localisation of germ plasm RNAs and Hermes protein in stage IV oocytes. When we injected full-length *nanos1* and *Xpat* mRNAs, we saw patterns of localisation similar to those at stage VI (Table 1). Some oocytes displayed Pattern I, with the RNAs co-localised exclusively with Hermes protein in a manner morphologically resembling germ plasm (Figure 6E,F). Hermes and *nanos1* RNA showed similar patterns when injected individually (Figure 6A). Staining mitochondria in vivo with the dye TMRE (see Machado et al. [22]) demonstrated that both protein and RNA were localised in association with mitochondrial aggregates, a defining property of germ plasm (Figure 2B). In contrast to stage VI oocytes, Pattern II localisation to germ plasm and to isolated granules lacking Hermes was found to be more prevalent (Figure 6E,F; Table 1), indicating that frequently both early and late pathways were active. Pattern III was also more common, but YFP-Hermes always localised to germ plasm. The inclusion of vitellogenin made no difference to these patterns, nor did destroying microtubules with colcemid.

Thus when analysed by confocal microscopy it is clear that Hermes protein, *nanos1* and *Xpat* RNAs frequently localised into germ plasm at mid oogenesis, but in some oocytes the late pathway fate was also followed, even by germ plasm RNAs.

### Sequence Requirements for Localisation of Nanos1 mRNA into Germ Plasm of Stage V/VI Oocytes

**Figure 7:**
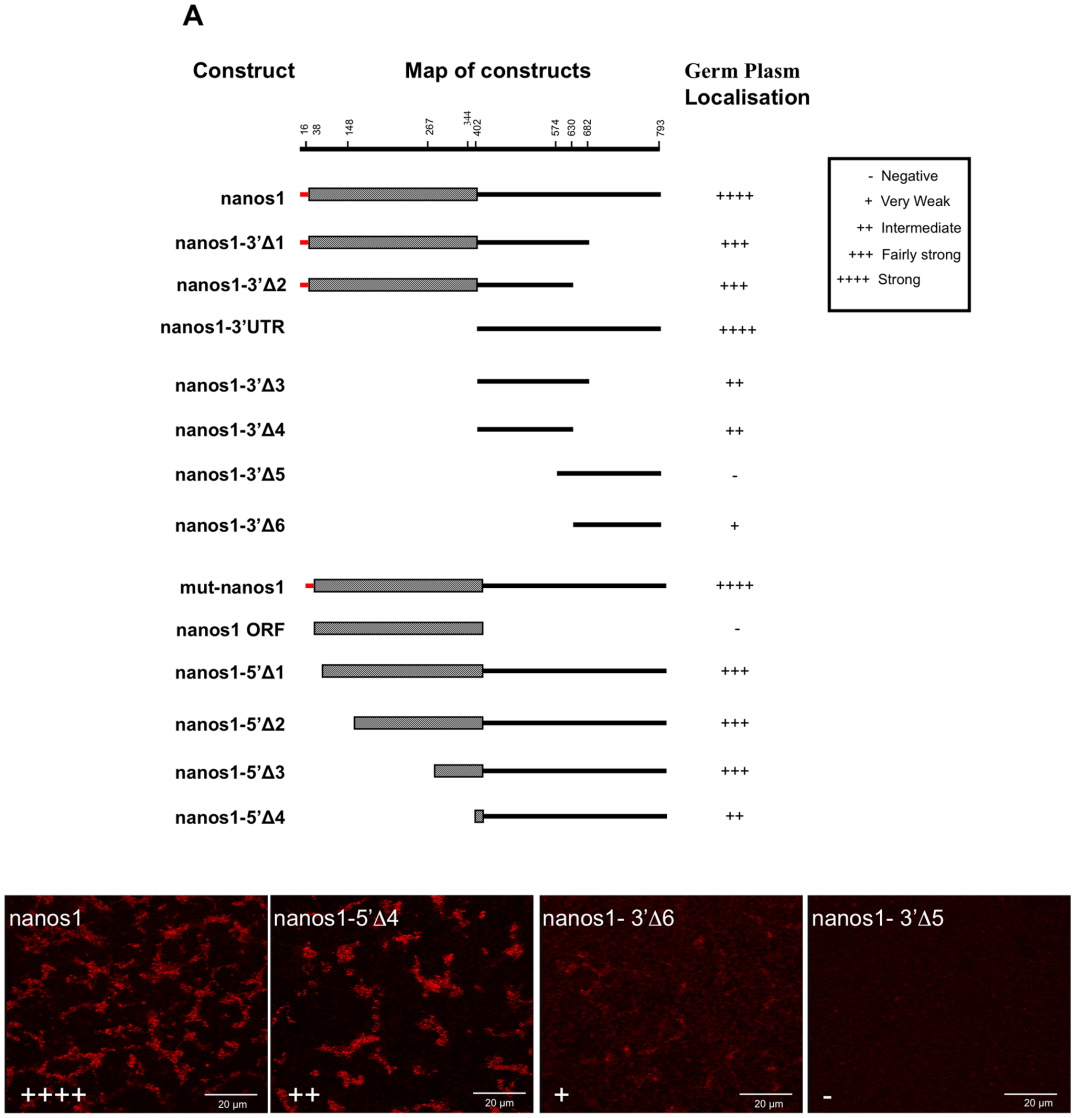
Localisation of nanos1 mutant transcripts in stage VI oocytes. 3′ and 5′ deletions used to create mutant transcripts are shown in schematic form (ORF, hatched; 5′ UTR, red; 3′ UTR, black). The map of the constructs is not drawn on a linear scale, but the residue numbers on the ruler correspond to the construct ends below. *mut-nanos1* consisted of the full length transcript with its start codon mutated to TTG. Oocytes were injected with Cy5 labelled mutant transcripts and examined after 48 h using constant gain settings on the confocal microscope. The subsequent localisation of the injected RNA was scored as plus or minus. Examples of the different degrees of localisation are shown below.

Other investigators have used deletion mutants to identify regions of the *nanos1* transcript that are needed for its localisation at early and mid stages of oogenesis. Given that in our experiments the RNA was apparently localised differently, we have tested a similar deletion series of *nanos1* mRNA in large oocytes (Figure 7). Zhou et al. [24] examined the localisation at stage IV by dissection of the oocytes followed by RNase protection assays. The authors were able to demonstrate that injected full-length *nanos1* RNA and its 3′UTR, localised to the vegetal cortex. However, the cortical localization of RNA transcripts corresponding to the first 150 and last 120 nucleotides of the nanos1 3′UTR, was equivalent to the entire 3′UTR. This localisation also required intact microtubules, as cold or nocadazole treatment prevented transport. Notably they found that injected *nanos1* RNA did not demonstrate cortical localisation in stage VI oocytes. In a related study Zhou et al. [32] tested localisation to the mitochondrial cloud of stage I oocytes using autoradiography of oocyte sections; this revealed that the first 250 nucleotides of the *nanos1* 3′UTR was required for localisation.

We have tested a similar deletion series to map those localisation signals in *nanos1* that mediate localisation in stage VI oocytes (Figure 7, Table 2). In spite of the apparent contradictory localisation we report, overall our observations on sequence requirements were similar to those seen by Zhou et al. [24] in stage IV oocytes, except that the sequences directed germ plasm localisation. This suggests that the final fate of the injected RNA is determined as much by the stage and nature of particular oocytes as by the details of the RNA sequence injected.

**Table 2:** Summary of localisation patterns displayed by injected mutant RNA.

	Number and proportion of oocytes displaying indicated localisation pattern.			
Injected RNA.	Pattern I (Germplasm only)	Pattern II (Germ plasm+late pathway)	Pattern III (latepathway only)	Negative
*nanos1 FL* (N = 16)	63/91 (69%)	19/91 (21%)	3/91 (3%)	6/91 (7%)
*nanos1-3′Δ1* (N = 5))	28/35 (80%)	0/35 (0%)	0/35 (0%)	7/35 (20%)
*nanos1-3′Δ2* (N = 4)	26/38 (68%)	0/38 (0%)	0/38 (0%)	12/38 (32%)
*nanos1-3′UTR FL* (N = 15)	57/90 (63%)	13/90 (14%)	5/90 (6%)	15/90 (17%)
*nanos1-3′Δ3* (N = 5)	27/31 (87%)	0/31 (0%)	0/31 (0%)	4/31 (13%)
*nanos1-3′Δ4* (N = 8)	46/58 (79.3%)	3/58 (5%)	0/58 (0%)	9/58 (16%)
*nanos1-3′Δ5* (N = 8)	0/50 (0%)	0/50 (0%)	0/50 (0%)	50/50 (0%)
*nanos1-3′Δ6* (N = 14)	46/102 (45%)	14/102 (14%)	3/102 (3%)	39/102 (38%)
*mut-nanos1* (N = 8)	22/38 (58%)	14/38 (37%)	0/38 (0%)	2/38 (5%)
*nanos1 ORF* (N = 5)	0/24 (0%)	0/24 (0%)	0/24 (0%)	24/24 (100%)
*nanos1-5′Δ1* (N = 3)	4/14 (29%)	4/14 (29%)	5/14 (36%)	1/14 (7%)
*nanos1-5′Δ2* (N = 4)	9/16 (56%)	3/16 (19%)	3/16 (19%)	1/16 (6%)
*nanos1-5′Δ3* (N = 3)	11/14 (79%)	3/14 (21%)	0/14 (0%)	0/14 (0%)
*nanos1-5′Δ4* (N = 4)	3/19 (16%)	0/19 (0%)	9/19 (47%)	7/19 (37%)

In each table cell the number of oocytes displaying a detectable signal with respect to the total number of injected oocytes is shown. The data summarised does not reflect the overall efficiency/strength of localisation by individual mutants, but this was scored as indicated in the key in Figure 7. N = the number of experiments using oocytes from different females. Cy5 or Cy3-labelled RNAs were routinely injected, with or without RNA encoding YFP-Hermes. FL; full length. mut-nanos1 is an RNA with a mutated initiation codon.

Kloc et al. [33] used electron microscope autoradiography of pre-vitellogenic stages to distinguish general Balbiani body localisation from that to the RNPs within it. At stage I the whole 3′UTR directed localisation to particles within the Balbiani body, but its proximal end (equivalent to our *nanos1-3′Δ3* & *Δ4* constructs) directed localisation to the Balbiani body, but not to the particles. Thus they identified an RNP targeting element in the distal 3′UTR, but only by the effect of its deficiency. We have directly tested an equivalent fragment, *nanos1-3′Δ6*, for its localisation ability at stage VI and found that it produced localisation into germ plasm particles at vitellogenic stages, although the overall frequency and strength of localisation was very poor (Figure 7, Table 2). On the other hand, the proximal end of the 3′UTR (*nanos1-3′Δ3* & *Δ4* ), directed efficient localisation into particles. Thus the overall function of fragments appears to be similar in our experiments and in those conducted by others at earlier stages, although the details of particle localisation are different.

Several studies have shown that vegetal localisation in mid oogenesis depends on multiple E2 and VM1 elements [48]–[50]. The proximal end of the 3′ UTR does contain these, but the distal 3′ region (*nanos1-3′Δ6*) contains only a single VM1 motif. Thus it is possible that quite different sequences are involved in localisation of this region of the RNA to germ plasm.

**Figure 8:**
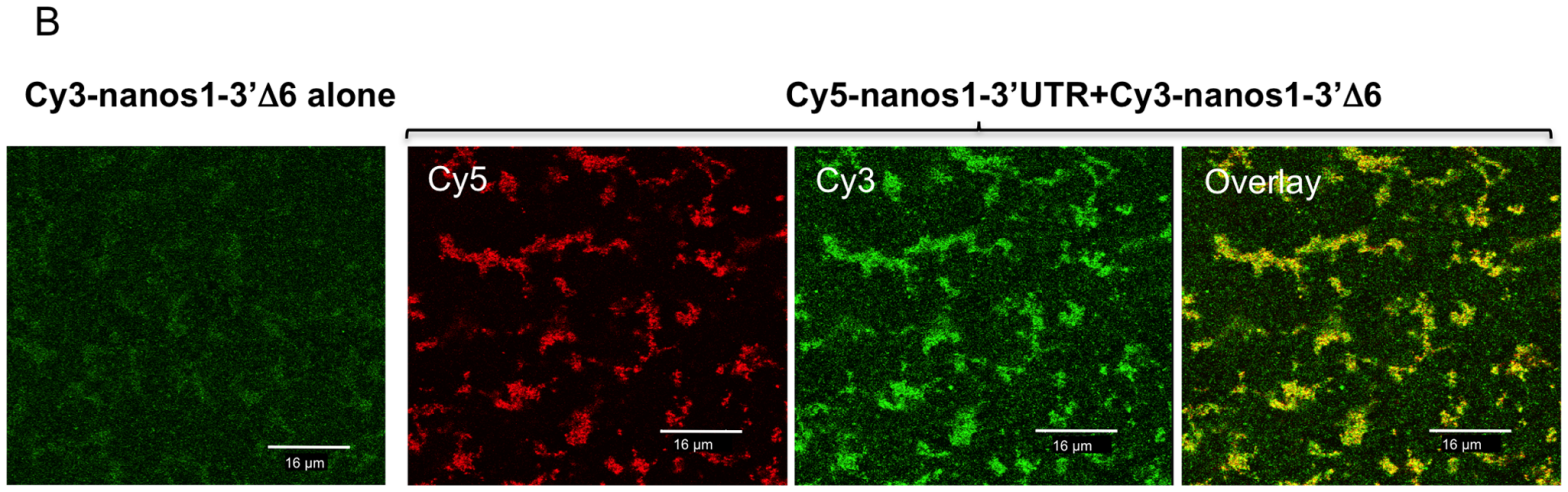
Co-operation between the mutant nanos1-3′Δ6 and the wild-type 3′UTR in germ plasm RNP localisation. Stage VI oocytes injected with Cy3-*nanos1 3′Δ6* RNA display a very weak localisation pattern after 48 h in culture. Localization of this RNA was greatly improved when co-injected with Cy5-labelled RNA consisting of the full length *nanos1* 3′UTR.

As mentioned earlier, Zhou et al. [24] were able to map sequences 150 nucleotides immediately adjacent to the open reading frame and an additional 120 nucleotides at the end of the 3′UTR, which in cis gave efficient localisation to the vegetal cortex in a manner identical to the full length 3′UTR. We were able identify a putative interaction between these two regions in experiments when full-length Cy5-*nanos1* 3′UTR was co-injected with Cy3-*nanos1-3′Δ6* RNA. Localisation of the Cy3-*nanos1-3′Δ6* mutant transcript (containing the last 163 nucleotides of the 3′UTR) was normally very weak, but was enhanced by co-injected *nanos1* 3′UTR (Figure 8). Similar results were obtained when labelled or unlabelled full-length *nanos1* or *nanos1 3′UTR* RNA were co-injected with Cy3-*nanos1-3′Δ6*. In four separate experiments 23/31 oocytes showed enhanced localisation of Cy3-*nanos1-3′Δ6* by co-injected full length *nanos1* RNA, and 23/30 showed enhancement by *nanos1-3′UTR* RNA. This cooperation in trans suggests that these two regions combine into a complex, either by binding to a common component, or by binding to components that multimerise.

### Localisation of Late Pathway RNAs Introduced into Oocytes

**Figure 9:**
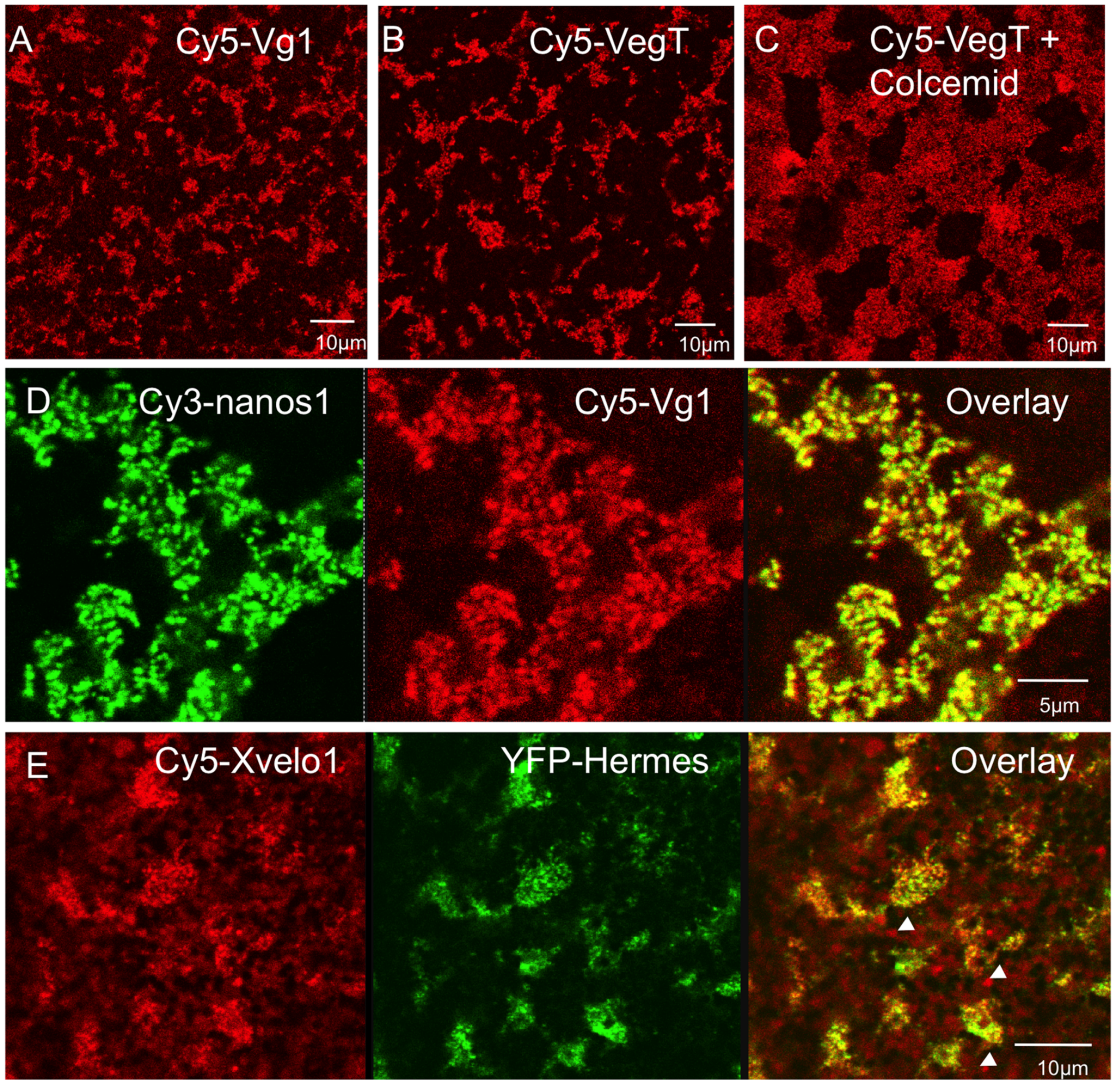
Localisation of non-germ plasm RNAs in stage V/VI oocytes. Cy5 or Cy3 labelled RNAs were injected into oocytes and examined 48 h later. In A–C and E vitellogenin was included in the culture medium. In panel F labelled *Xvelo1* full length RNA was co-injected with RNA encoding YFP-Hermes protein. The localised RNA or protein detected is indicated in each panel. In panel F germ plasm aggregates are indicated with arrows and late pathway particles with arrowheads.

*Xvelo1* mRNA has been reported to have a vegetal localisation pattern like late pathway RNAs, being excluded from the Balbiani body at early stages, and found in the general vegetal cortex at late stages [51]. Surprisingly it lacks sequences typical of other vegetally localised RNAs, although it binds similar proteins. The zebrafish homologue of Xvelo1 is Bucky ball (buc), which is necessary for oocyte polarisation and germ plasm formation in this species [39], [40]. In contrast to *Xenopus Xvelo1*, *buc* RNA is localised to the Balbiani body at early stages and to the animal pole at late stages, before becoming unlocalised in later oogenesis. In *Xenopus* there are two splice variants, differing in their internal ORF exon structure, but not in their UTRs [51]. When microinjected into stage V/VI oocytes Cy5-labelled *Xvelo1* long form (FL) was localised to germ plasm, but in some instances there were also isolated particles (arrowheads in Figure 9F), separate from the germ plasm islands, indicative of Pattern II (Table 1). Within the islands *Xvelo1* RNA co-localised with YFP-Hermes, but Hermes was absent from the isolated particles, which would therefore be expected to be of the late pathway type. Thus, in our experiments *Xvelo1FL* RNA localised to both late pathway and germ plasm particles in large oocytes, that is it gave Patterns I and II.

*Vg1* and *VegT* are two endogenously expressed RNAs localised by the late pathway. However, in our experiments injected Cy5-*Vg1* RNA localised in a combination of Patterns I, II and III in large oocytes, and *VegT* by Pattern I (Table I). This was true whether vitellogenin was present or absent and it was also true when microtubules were dissociated with colcemid (Figure 9A–F;). The Cy5-*Vg1* RNA was coincident with both YFP-Hermes and Cy3-*nanos1* in the germ plasm particles (Figure 9D,E) and Cy5-*VegT* also co-localised with YFP-Hermes (not shown). Thus in our experiments, the late pathway RNAs localise wholly or substantially into germ plasm particles in large oocytes.

Localisation of injected RNAs is normally studied in stage I–IV, early to mid-oogenesis oocytes. As explained earlier we found several patterns of germ plasm RNA distribution in stage IV oocytes, but the oocytes we happened to use in the relatively small number of experiments on late pathway RNAs showed exclusive localisation of *nanos1* and *Xpat* RNAs to germ plasm (Pattern I). Figure 6 summarises experiments showing that, in these experiments, *nanos1*, *VegT* and *Vg1* RNAs localised substantially into germ plasm in these stage IV oocytes. The latter two RNAs co-localised with the germ plasm RNA *nanos1* (Figure 6C,D). This indicates that in our hands injected *Vg1* and *VegT* RNAs can localise significantly into germ plasm from mid to late oogenesis.

### Continuity of Localised Injected RNAs from Oocyte to Egg Germ Plasm

The oocyte germ plasm islands labelled with injected Cy5-RNA or YFP-Hermes are very large compared to the endogenous islands previously reported in fertilized eggs, using lower resolution imaging after in situ hybridisation [52], [53], or antibody staining at higher resolution [22]. In this section we seek to demonstrate that the substantial germ plasm islands in the oocyte progress to sparser zygotic structures. Incidentally this will confirm that the structures we identify as germ plasm in the oocyte are continuous with the well-known smaller definitive germ plasm islands of the egg. In the experiments described above we have focused on Hermes protein as a germ plasm marker in oocytes, but Hermes protein has been reported to be lost during oocyte maturation [41], [42]. However, these reports used western blotting to quantify the protein and, since only a fraction of Hermes was reported to be present in germ plasm, it was possible that it persists in germ plasm after maturation.

**Figure 10:**
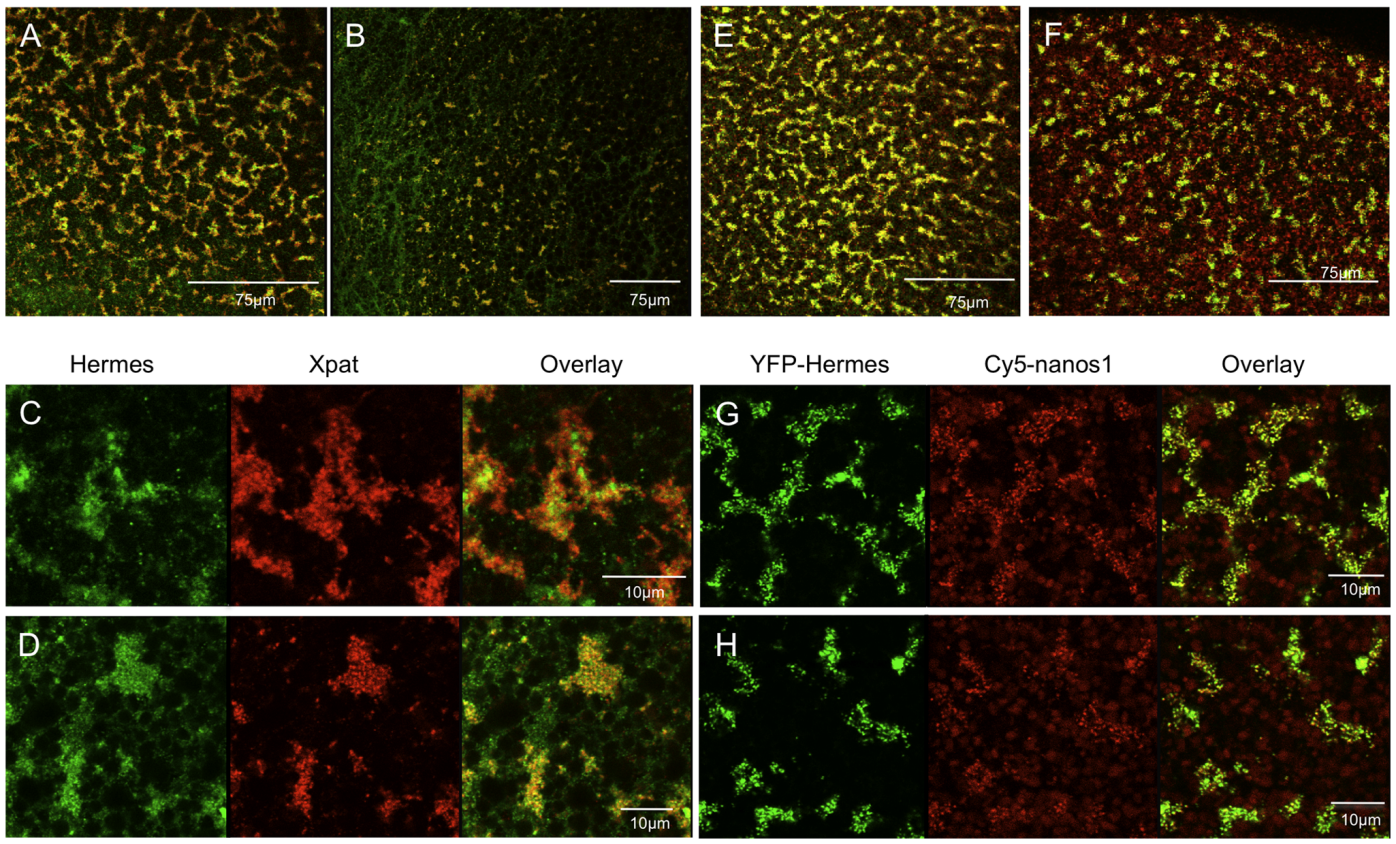
The behaviour of endogenous and exogenous germ plasm molecules during oocyte maturation. A–D. Fixed oocytes stained with antisera against Hermes (green) and Xpat (red). E–H. Live oocytes expressing YFP-Hermes (green) and injected Cy5-*nanos1* (red). A. The wide field of large islands in a control oocyte; the overlay of Hermes and Xpat is shown. B. A similar view of newly fertilised eggs showing the dramatic reduction in fluorescent islands. C. Detail of islands in a control oocyte. D Detail of a fertilised egg. Note that Hermes and Xpat are in distinct particles. E. The wider field of YFP-Hermes coincident with Cy5-*nanos1* is similar to the endogenous molecules in A. F. When these oocytes were matured with progesterone the field was reduced, as in B, but less so; this is expected, since fertilised eggs have progressed further. G,H. Details of the fields in E and F respectively.

When we compared oocytes and fertilised eggs stained with Hermes and Xpat antisera a remarkable reduction in the amount of germ plasm was observed (Figure 10A, B). The individual islands were sparser and smaller but, as in oocytes, Hermes and Xpat protein were present in distinct particles (Figure 10C, D). To establish what happened to the structures labelled by Cy5-RNA and YFP-Hermes during maturation, oocytes were co-injected with Cy5-*nanos1* and *YFP-Hermes* mRNA and allowed to mature in vitro after adding progesterone. The matured oocytes show a reduction in the number and size of germ plasm islands compared to controls (Figure 10E–H). The reduction is not quite as great as in the stained fertilized eggs, but of course oocytes matured in vitro have only progressed part of the way to the fertilized egg state.

Thus the injected RNA and co-expressed Hermes protein parallel the behaviour of endogenous molecules in unmanipulated oocytes and eggs. It is clear that Hermes persists in germ plasm, and that there is a considerable decrease in the amount of germ plasm during maturation and egg laying. This loss of germ plasm-associated components is further highlighted by a reported decrease in the amounts of *Xpat* mRNA in Northern blots of oocytes and eggs [29].

## Discussion

In the experiments described here we find that within the cortex labelled germ plasm RNAs injected into large *Xenopus* oocytes usually localise into germ plasm RNP particles, where they co-localise with the RNA-binding protein Hermes. In addition to the germ plasm a proportion of the RNA may also localise into late pathway particles, or in occaisionally the RNA may even display an exclusive late pathway fate. We have previously reported that the protein Poc1B (formerly called xPix2) also localises into germ plasm [35], where it is coincident with the protein Xpat [35], and we show here that Xpat particles, an established germ plasm marker [22], are different from those containing Hermes and injected germ plasm RNAs, although the two are closely interspersed. We confirm the identity of the aggregated particles as germ plasm by: (i) their co-concentration with mitochondria, a major germ plasm constituent; (ii) the fact that they contain the germ plasm protein Hermes; (iii) that they are associated with other particles containing the germ plasm protein Xpat; and (iv) their continuity into the kind of structure in eggs previously identified as definitive germ plasm. The association with mitochondria is particularly important because mitochondrial aggregates in the vegetal cortex are regarded as diagnostic of germ plasm. In reality the identification of most other germ plasm components was originally dependent on this association.

By elimination, the mechanism by which the RNA and protein is localised appears to be diffusion and entrapment, since it is not dependent on the integrity of microtubules, microfilaments or cytokeratins, within the time scale of the experiments here. The mechanism is thus identical to that previously identified in previtellogenic oocytes, where it was concluded that the RNAs are localised into RNP particles of the *Xenopus* Balbiani body by diffusion and entrapment [19], [20]. This also appears to be a conserved process utilised by *nanos* mRNA to localise into the germ granules of *Drosophila* [21]. However, the conclusion that localisation occurs independently of microtubules in previtellogenic *Xenopus* oocytes has not gone unchallenged. Heinrich et al. [54] showed that the rate of localisation to the Balbiani body was dependent on the ATP pool and on kinesin II. It is possible that the rate of localisation was subtly changed when we removed microtubules. The latter might affect the rate of localisation in indirect ways, for example through cytoplasmic streaming, as in the *Drosophila* oocyte, or in *C. elegans*, where it is actin dependent [55], [56]. What is clear from our experiments, as with those performed on stage I oocytes, is that diffusion is sufficient to enable localisation.

Our data make it likely that Hermes and the injected RNAs do not co-migrate during localisation because, when labelled RNA and mRNA encoding Hermes are co-injected into the equator, the translated protein diffuses faster across the oocyte than the labelled RNA. This contrasts with Xpat, which forms fields of ectopic particles in the absence of vitellogenin in the external medium and requires microtubules for transport to the vegetal pole [22]. We would hypothesise that Xpat needs microtubules because it first forms particles, which would diffuse very slowly. Conversely Hermes protein and the RNAs studied here enter particles only at their final destination, diffusing to get there. This model fits with the observation that newly expressed Xpat protein does not enter pre-existing germ plasm particles [22].

Our observation of RNA localisation into cortical particles at late stages of oogenesis is at odds with the original report of Yisraeli and Melton [23], but it has since been reported by Kloc et al. [27] that *Vg1* mRNA localises into late pathway granules in larger oocytes. Since most localisation studies have focused on early and mid oogenesis, we also looked at the localisation of *nanos1* and *Xpat* RNAs in stage IV oocytes. In most of our oocyte batches the RNAs were localised into germ plasm granules, as seen at stage VI. However, oocytes from several other females of a different cohort in our colony displayed isolated Cy5-labelled RNPs that lacked Hermes protein, presumably late pathway structures, in addition to a typical germ plasm RNA localisation pattern. Because exclusive late pathway localisation was expected from the literature, we looked for differences in our methods from those used by others, who have reported localisation of early RNAs by the late pathway. However, the behaviour of labelled RNA and protein was not affected by changes in the experimental conditions employed (disrupting the cytoskeleton, presence/absence of vitellogenin, presence/absence of follicle and thecal cells).

The localisation of the *nanos1-*3′UTR construct was inefficient and highly variable in both stage VI and stage IV oocytes. Again this appeared to be an oocyte batch-sensitive issue and the UTR-only data are skewed by the fact that in the initial part of this work the UTR was localising to late pathway particles, while full-length *nanos1* was consistently and robustly localising to germ plasm. The more ambiguous localisation of the 3′UTR-only construct emphasises that the final fate of the RNA is finely balanced in injection experiments. It seems that the coding region (ORF) has a substantial influence on localisation to germ plasm, but the ORF itself is not localised within the oocyte.

Although our data support the contention, discussed in the Introduction, that the distinction between the early and late pathways is less absolute than originally thought, we clearly find that germ plasm RNAs can localise into germ plasm robustly at late stages (Table 1). The sequences required for this process were similar to those previously found for localisation into RNPs in the Balbiani body [32], [33]. It should be mentioned that the majority of earlier studies were reliant on relatively low resolution in situ methods. Given the high density of oocyte (as opposed to egg) germ plasm structures reported here, some of these earlier reports might not have distinguished early and late pathway localisation, particularly if both occurred together, as seen in Pattern II.

However it may be that oocytes from frogs in other labs differ from ours, and we ourselves have found differences between animals, particularly with regard to the nanos1-3′UTR construct. One possibility that may explain this variation is that some oocytes contain spare capacity to form ectopic RNP particles. These would require cytoskeletal transport, just like Xpat protein particles [22]. However, this may be lacking in oocytes from many of the females that we have used, so diffusion of molecules into particles at the final destination becomes the default state and predominates.

When we examined the behaviour of RNAs naturally localised by the late pathway the results were also surprising. *Xvelo1* is a *Xenopus* RNA that is excluded from the Balbiani body at early stages, and localised to the vegetal cortex at late stages, both classic features of late pathway RNAs [51]. In zebrafish, the homologous RNA, Bucky ball, is a mitochondrial cloud constituent, as probably is its protein [39], [40]. We have found that in *Xenopus Xvelo1* protein is a constituent of the Balbiani body and germ plasm at all stages (in preparation). Xvelo1 mRNA is unusual because it does not have conventional late pathway sequences, although it and the *Vg1* localisation element bind similar proteins [51]. We found that labelled *Xvelo1* RNA localises into germ plasm or jointly into germ plasm and late pathway granules. The differences in the localisation patterns observed may be because the in situ methods originally used to identify endogenous *Xvelo1* transcripts [51] were not high enough resolution to unambiguously distinguish between localisation to germ plasm and late pathway structures at the vegetal cortex. As mentioned earlier, differences between animal colonies may cause variability, and this is difficult to control for.

Our results with the injected definitive late pathway RNAs *VegT* and *Vg1* were more surprising, since we found them to be substantially localised into germ plasm structures. However, it must be noted that this was in oocytes that exclusively localised *nanos1* RNA into germ plasm. Unfortunately, we cannot predict how oocytes behave, so those with the mixed localisation pattern were not tested. Overall our experiments fit with the view that the localisation pathways are only subtly different.

What, one might ask, is the biological significance of a process which localises RNAs into germ plasm, or sometimes late pathway RNPs, by a cytoskeleton-independent mechanism, as previously seen in earlier Balbiani body stages? [19], [20]. That diffusion is sufficient for localisation in injection experiments does not exclude a role for the cytoskeleton in constructing localised structures, since the organisation of the vegetal cortex must depend on the prior action of the cytoskeleton, as does the whole polarisation of the oocyte. It was clearly evident that disruption of the cytoskeleton resulted in a corresponding alteration of germ plasm structure because, in the absence of intact microtubules, the germ plasm islands ran together. However, the injected RNAs and Hermes still entered localised RNP particles efficiently, and these RNPs were still aggregated in the cortex. It seems likely that the reason there is localisation of exogenous molecules is either that there is continuous exchange of the RNP components with free molecules in the cytoplasm, or that there is spare capacity to add to them from an injected pool. When we expressed YFP-Hermes and then stained the oocyte for total Hermes, all antiserum-identified particles were fluorescent, so we conclude a new set of particles is not formed (data not shown). Moreover, the fluorescence patterns from individual RNAs and protein is identical to those in combination, so there must be spare capacity to incorporate all the individual molecules into localised RNPs, as was concluded for *nanos* RNA in *Drosophila* [21]. Alternatively, a scenario may exist in which both endogenous and injected RNAs are continually remodelled into new particles, i.e. all particles are always new! We show by FRAP that YFP-Hermes is incorporated into endogenous RNP granules by exchange from the soluble pool of newly translated YFP-Hermes protein. Thus, what the fluorescent molecules reveal is the pre-existing distribution of exchangeable RNP structures in the oocyte. These are found not only in the cortex, but within the internal cytoplasm, as previously seen at earlier stages [29], [44]. It is not clear if these internal particles normally contain RNAs in uninjected oocytes, but we will show elsewhere that a number of RNP constituent proteins, including Hermes and Xvelo1, localise into these internal particles, individually and in combination. This argues that the internal particles exist in unmanipulated oocytes, rather than their being created de novo following micro-injection.

One might debate how far one is looking at an artificial situation by injecting large numbers of molecules at one time, when they are normally made over a prolonged period, and in the case of RNAs they are transcribed and processed within the nucleus, then transported to the cytoplasm. While it is quite possible that RNAs exported from the nucleus wholly, or in part, localise by diffusion, in *Drosophila* it has been shown that *oskar* mRNA derived from intronless genes localises to germ plasm only if accompanied by *oskar* RNA that has been through a splicing process [57]. Whether introns play a comparable role in any *Xenopus* RNA localisation is unclear. However in *Drosophila nanos* mRNA is localised by diffusion/entrapment [21], revealing that the diffusion process has some generality, although the possibility that splicing of the normal *nanos* gene was also involved was not excluded. Nevertheless, in *Xenopus* nucleus-independent processes are likely to be important because many late pathway RNAs are dispersed throughout the oocyte, prior to later localisation. This property is not restricted to late RNAs, since this is also a feature of some RNAs which ultimately localise to germ plasm.

Since components apparently diffuse in and out of germ plasm particles, as we show for Hermes, one could argue that at the very least diffusion plays a role in particle maintenance. In *C. elegans* P-granules are very dynamic and appear to dissolve and reform continuously. It seems that their localisation depends more on localised assembly/disassembly than on transport, with assembly being favoured in the germline lineage [58], [59]. Perhaps this is also true of *Xenopus*.

What is the significance of the internal particles? One can envisage a facilitated diffusion process from nucleus to vegetal cortex in which proteins and RNAs move by a sort of “pass the parcel” mechanism. If the RNA is incorporated more stably into structures at the vegetal cortex than to those found internally, there would be a net diffusion towards the vegetal pole – in the analogy the “parcels” would end up with selfish individuals that do not hand them on. This fits our observation that Hermes protein diffuses in and out of the cortical particles, but *nanos1* RNA does not do so, at least on the short timescale of a FRAP experiment. This model would produce net targeted diffusion of RNA, but proteins would be maintained in internal particles, as well as at the cortex. Two predictions of this model are that internal particles bind RNAs less stably and that they would have a different composition from those in cortical germ plasm islands. The latter is more easily testable. It should be mentioned that, even in *Drosophila*, the transport of Oscar-containing particles via micro-tubules has only a very small bias towards the posterior pole of otherwise random, kinesin-based movement, yet the result is tight localisation [60]. We can find no evidence for distinct kinds of internal cytoplasmic particles (e.g. all those labelled with Cy5-*nanos1* are also labelled by YFP-Hermes; not shown). It is therefore possible that the distinction between the germ plasm and late pathway compartments occurs only at the cortex.

One striking incidental result of our study is the demonstration that the amount of germ plasm decreases radically in the progress from oocyte to egg via maturation. The significance of this is unclear. In *C. elegans* mislocalised P granules and their components are destroyed [61]–[63], an essential part of the mechanism to restrict their function to the germline [58]. It is conceivable that a similar process operates in *Xenopus* to refine the distribution of germ plasm, and like its counterpart in *C. elegans*, germ plasm in *Xenopus* is much more labile than previously supposed [58], [59]. Our observation of RNA and protein diffusion into the germ plasm particles is certainly consistent with this view.

## Materials and Methods

### Plasmid Contructs and in vitro RNA Transcription

Capped mRNA was synthesised in vitro using linearised plasmid templates and the Message Machine in vitro transcription kit (Ambion). To prepare fluorescently labelled transcripts for microinjection Cy5 or Cy3-UTP (GE Healthcare) or Aminoallyl-UTP - ATTO-390 (JENA Bioscience) was included in the transcription reaction.

Full length sense mRNA for *nanos1* was synthesised, using T7 RNA polymerase from the plasmid pSPORT1 linearised with BamHI [64]. The nanos1 3′UTR was transcribed using SP6 RNA polymerase from the plasmid pCS2+ linearised with XhoI [20]. Sense *Vg1* RNA was transcribed using SP6 RNA polymerase from the plasmid pSP64TVg1 linearised with XbaI [65]. Full length *Xpat* RNA was transcribed with T3 RNA polymerase from the plasmid pBSK linearised with XhoI [29]. Sense *VegT* transcripts were synthesised using SP6 RNA polymerase from pBratCS2 linearised with NotI.

Sense *Xvelo1* mRNA was transcribed using T3 RNA polymerase from the plasmid Xvelo1-MC118 (in pBK-CMV) linearised with XhoI (provided by T. Pieler).

The ATG codon in the *nanos1* (pSPORT1) plasmid was mutated to TTG to prepare the mutant *nanos1* (*mut-nanos1*) construct using the Stratagene site directed mutagenesis kit and the primers 5′-GAACAATTCCAACTTGGATGGCGGTC-3′ and 5′-GACCGCCATCCAAGTTGGAATTGTTC-3′. RNA was transcribed using T7 RNA polymerase following linearisation with BamHI.

The *nanos1*-ORF construct was prepared by amplifying the coding region in *nanos1* (pSPORT1), with the following primers 5′-AAAAGATCTATGGATGGCGGTCTCTGCTTTGAC-3′and 5′-AGAATTCTCAGTGTCTCAGCTTTGGGTTATTAC-3′. The product was digested with BglII and EcoRI and ligated into the corresponding sites in pSPJC2L. RNA was transcribed using SP6 RNA polymerase following linearisation with XhoI. YFP-Hermes in pCS2+ was transcribed with SP6 RNA polymerase following linearisation with NotI.

### nanos1 5′ Deletion Constructs

*Xenopus nanos1* 5′ deletion mutants were constructed by PCR using the nanos1 (pSPORT1) plasmid as a template. For *nanos1-5′Δ1* the primers 5′-AAAGAATTCACTCATGGAGCGACTACTTG-3′ and 5′-CACGCGTACGTAAGCTTGGATCCTCTAG-3′ were used to amplify nucleotides 38–793 of the *nanos1* cDNA; *nanos1-5′Δ2* primers 5′-AAAGAATTCCGTTACCCAGTAATGAGTC-3′ and 5′-CACGCGTACGTAAGCTTGGATCCTCTAG-3′ amplified nucleotides 148–793; *nanos1-5′Δ3* primers 5′-AAAGAATTCCAGTACTAAGAGGCTACAC-3′ and 5′-CACGCGTACGTAAGCTTGGATCCTCTAG-3′ amplified nucleotides 267–793 and for *nanos1-5′Δ4*, primers 5′-AAAGAATTCCGTCGGCTCCTCAGAGAT-3′ and 5′-CACGCGTACGTAAGCTTGGATCCTCTAG-3′ were used to amplify nucleotides 344–793.

Amplified products were then ligated into pGEM-T Easy. All constructs were linearised with BamHI, and RNA transcribed with SP6 RNA polymerase, except for *nanos1-5′Δ3* where T7 RNA polymerase was used.

### nanos1 3′ Deletion Constructs

Deletion mutants *nanos1-3′Δ1*, *nanos1-3′Δ3*, *nanos1-3′Δ5* and *nanos1-3′Δ6* were constructed by PCR using the nanos1 pSPORT1 plasmid as a template. For *nanos1-3′Δ1* the primers 5′-AAAAGATCTCAGAACAATTCCAACATGGATG-3′-3′ and 5′-AAAGAATTCTCTGGAGAGCTGGAATGTCCGG-3′ were used to amplify nucleotides 1–682 of the nanos1 cDNA; *nanos1-3′Δ3* primers 5′-AAAAGATCTCTGAACTGGAAGGAACCCTGGG-3′ and 5′-AAAGAATTCTCTGGAGAGCTGGAATGTCCGG-3′ amplified nucleotides 399–682; *nanos1-3′Δ5* primers 5′-AAAAGATCTGATGTCTCACTCGGCACCCACG-3′ and 5′-AAAGAATTCTGACGTCGCATGCACGCGTACG-3′ amplified nucleotides 574–793; *nanos1-3′Δ6* primers 5′-AAAAGATCTGGGGCCAAGCGGTGCCCATCCC-3′ and 5′-AAAGAATTCTGACGTCGCATGCACGCGTACG-3′ amplified nucleotides 631–793.

Amplified products were digested with BglII and EcoRI and ligated into the corresponding sites in pSPJC2L. *nanos1-3′Δ2* was prepared by digesting the *nanos1* pSPORT1 plasmid with SmaI to release the first 630 bp of the nanos1 cDNA. The SmaI fragment was ligated into EcoRI digested/blunted pSPJC2L. *nanos1-3′Δ4* was prepared by digesting the nanos1-3′UTR construct in pCS2+ with BamHI and SmaI, releasing an insert corresponding to nucleotides 402–630 of *nanos1*. The insert was blunted and ligated into EcoRI-digested/blunted pSPJC2L. For sense RNA all constructs were linearized with XhoI and RNA transcribed with SP6 RNA polymerase.

### Culture and Injection of Oocytes

Oocytes of *Xenopus laevis* (Daudin) were obtained by manual dissection from ovaries if they were to be cultured within intact follicles, or by collagenase treatment for isolation without follicle cells [20]. The former retain intact layers of follicle and theca cell layers. They were cultured in oocyte culture medium (OCM) [23], with or without 10% vitellogenin-containing frog serum, as previously described [22]. They were microinjected with RNA in 27.6 nl of water Using a Drummond imicroinjection system. After 24–72 h they were either examined live in OCM by confocal microscopy, or fixed in methanol containing 1% HCOOH at −20°C overnight, or alternatively in 2% HCOOH in PBS at 4°C.

To disrupt microtubules, oocytes were cultured in OCM containing 5 µg/ml colcemid (Sigma). Actin polymerisation was disrupted by incubating oocytes in 1 µg/ml cytochalasin D (Sigma). Incubation of injected/uninjected oocytes in inhibitors was for 24–48 hours at 21°C.

Oocytes were matured in vitro by addition of 125 µg/ml progesterone. Maturation was scored by the appearance of the germinal vesicle (white spot formation) at the animal pole of the oocyte. Eggs were obtained and handled as described previously [22]. The work was carried out under a UK Home Office-approved animal procedures project licence and approved by the University of Warwick Biological Ethics Committee.

### Microscopy and Staining

Oocytes were stained and analyzed with antibodies as described previously [22]. Rabbit anti-Hermes was a kind gift of Dr Malgorzata Kloc and the late Dr Larry Etkin. Sheep Xpat antiserum was affinity purified as described by Machado et al. [22]. Staining of Cytokeratin filaments was performed with the monoclonal anti-pan cytokeratin clone C-11 antibody (Sigma). α-Tubulin was detected with the polyclonal antibody #2144 (Cell Signaling Technology). Microscopy was conducted with a Leica SP2 confocal microscope, using a standard vertical set up and the oocytes were held in 0.7 mm deep chambers under coverslips.

## References

[1] Macdonald PM (2011) mRNA localization: assembly of transport complexes and their incorporation into particles. Current Opinion in Genetics & Development 21: 407–413. 21536427 10.1016/j.gde.2011.04.005 PMC4301680

[2] Palacios IM, St Johnston D (2001) Getting the message across: the intracellular localization of mRNAs in higher eukaryotes. Annu Rev Cell Dev Biol 17: 569–614. 11687499 10.1146/annurev.cellbio.17.1.569

[3] King ML, Messitt TJ, Mowry KL (2005) Putting RNAs in the right place at the right time: RNA localization in the frog oocyte. Biology of the Cell 97: 19–33. 15601255 10.1042/BC20040067

[4] Zhou Y, King ML (2004) Sending RNAs into the future: RNA localization and germ cell fate. Iubmb Life 56: 19–27. 14992376 10.1080/15216540310001658886

[5] Yaniv K, Yisraeli JK (2001) Defining cis-acting elements and trans-acting factors in RNA localization. International Review of Cytology - a Survey of Cell Biology, Vol 203 203: 521–539. 10.1016/s0074-7696(01)03015-7 11131525

[6] Kloc M, Bilinski S, Chan AP, Allen LH, Zearfoss NR, et al (2001) RNA localization and germ cell determination in *Xenopus* . International Review of Cytology - a Survey of Cell Biology, Vol 203 203: 63–91. 10.1016/s0074-7696(01)03004-2 11131528

[7] Gagnon JA, Mowry KL (2011) Visualization of mRNA localization in *Xenopus* oocytes. Methods Mol Biol 714: 71–82. 21431735 10.1007/978-1-61779-005-8_5 PMC3181151

[8] Kloc M, Dougherty MT, Bilinski S, Chan AP, Brey E, et al (2002) Three-dimensional ultrastructural analysis of RNA distribution within germinal granules of *Xenopus* . Developmental Biology 241: 79–93. 11784096 10.1006/dbio.2001.0488

[9] Heasman J, Quarmby J, Wylie CC (1984) The mitochondrial cloud of *Xenopus* oocytes - the source of germinal granule material. Developmental Biology 105: 458–469. 6541166 10.1016/0012-1606(84)90303-8

[10] Kloc M, Bilinski S, Dougherty MT (2007) Organization of cytokeratin cytoskeleton and germ plasm in the vegetal cortex of *Xenopus laevis* oocytes depends on coding and non-coding RNAs: Three-dimensional and ultrastructural analysis. Experimental Cell Research 313: 1639–1651. 17376434 10.1016/j.yexcr.2007.02.018 PMC2613015

[11] Heasman J, Wessely O, Langland R, Craig EJ, Kessler DS (2001) Vegetal localization of maternal mRNAs is disrupted by VegT depletion. Developmental Biology 240: 377–386. 11784070 10.1006/dbio.2001.0495

[12] Kloc ME, Etkin LD (1994) Delocalisation of Vg1 messenger RNA from the vegetal cortex in *Xenopus* oocytes after destruction of *Xlsirt* RNA. Science 265: 1101–1103. 7520603 10.1126/science.7520603

[13] Cox RT, Spradling AC (2003) A Balbiani body and the fusome mediate mitochondrial inheritance during Drosophila oogenesis. Development 130: 1579–1590. 12620983 10.1242/dev.00365

[14] Pepling ME, Wilhelm JE, O'Hara AL, Gephardt GW, Spradling AC (2007) Mouse oocytes within germ cell cysts and primordial follicles contain a Balbiani body. Proceedings of the National Academy of Sciences of the United States of America 104: 187–192. 17189423 10.1073/pnas.0609923104 PMC1765432

[15] Tao QH, Yokota C, Puck H, Kofron M, Birsoy B, et al (2005) Maternal Wnt11 activates the canonical wnt signaling pathway required for axis formation in Xenopus embryos. Cell 120: 857–871. 15797385 10.1016/j.cell.2005.01.013

[16] Ku M, Melton DA (1993) Xwnt-11 - a maternally expressed *Xenopus Wnt* gene. Development 119: 1161–1173. 8306880 10.1242/dev.119.4.1161

[17] Schroeder KE, Condic NL, Eisenberg LM, Yost HJ (1999) Spatially regulated translation in embryos: Asymmetric expression of maternal Wnt-11 along the dorsal-ventral axis in *Xenopus* . Developmental Biology 214: 288–297. 10525335 10.1006/dbio.1999.9426

[18] Alarcon VB, Elinson RP (2001) RNA anchoring in the vegetal cortex of the *Xenopus* oocyte. Journal of Cell Science 114: 1731–1741. 11309203 10.1242/jcs.114.9.1731

[19] Kloc M, Larabell C, Etkin LD (1996) Elaboration of the messenger transport organizer pathway for localization of RNA to the vegetal cortex of *Xenopus* oocytes. Developmental Biology 180: 119–130. 8948579 10.1006/dbio.1996.0289

[20] Chang P, Torres J, Lewis RA, Mowry KL, Houliston E, et al (2004) Localization of RNAs to the mitochondrial cloud in *Xenopus* oocytes through entrapment and association with endoplasmic reticulum. Molecular Biology of the Cell 15: 4669–4681. 15292452 10.1091/mbc.E04-03-0265 PMC519158

[21] Forrest KM, Gavis ER (2003) Live imaging of endogenous RNA reveals a diffusion and entrapment mechanism for nanos mRNA localization in *Drosophila* . Current Biology 13: 1159–1168. 12867026 10.1016/s0960-9822(03)00451-2

[22] Machado RJ, Moore W, Hames R, Houliston E, Chang P, et al (2005) *Xenopus* Xpat protein is a major component of germ plasm and may function in its organisation and positioning. Developmental Biology 287: 289–300. 16216237 10.1016/j.ydbio.2005.08.044

[23] Yisraeli JK, Melton DA (1988) The maternal messenger RNA Vg1 is correctly localized following injection into *Xenopus* oocytes. Nature 336: 592–595. 3200307 10.1038/336592a0

[24] Zhou Y, King ML (1996) RNA transport to the vegetal cortex of *Xenopus* oocytes. Developmental Biology 179: 173–183. 8873762 10.1006/dbio.1996.0249

[25] Betley JN, Heinrich B, Vernos I, Sardet C, Prodon F, et al (2004) Kinesin II mediates Vg1 mRNA transport in *Xenopus* oocytes. Current Biology 14: 219–224. 14761654 10.1016/j.cub.2004.01.028

[26] Yisraeli JK, Sokol S, Melton DA (1990) A 2-Step model for the localization of maternal messenger-RNA In *Xenopus* oocytes - Involvement of microtubules and microfilaments in the translocation and anchoring of Vg1 messenger RNA. Development 108: 289–298. 2351071 10.1242/dev.108.2.289

[27] Kloc M, Wilk K, Vargas D, Shirato Y, Bilinski S, et al (2005) Potential structural role of non-coding and coding RNAs in the organization of the cytoskeleton at the vegetal cortex of *Xenopus* oocytes. Development 132: 3445–3457. 16000384 10.1242/dev.01919

[28] Allen L, Kloc M, Etkin LD (2003) Identification and characterization of the Xlsirt cis-acting RNA localization element. Differentiation 71: 311–321. 12919101 10.1046/j.1432-0436.2003.7106003.x

[29] Hudson C, Woodland HR (1998) *Xpat*, a gene expressed specifically in germ plasm and primordial germ cells of *Xenopus laevis* . Mechanisms Of Development 73: 159–168. 9622619 10.1016/s0925-4773(98)00047-1

[30] Choo S, Heinrich B, Betley JN, Chen Z, Deshler JO (2005) Evidence for common machinery utilized by the early and late RNA localization pathways in *Xenopus* oocytes. Developmental Biology 278: 103–117. 15649464 10.1016/j.ydbio.2004.10.019

[31] Claussen M, Horvay K, Pieler T (2004) Evidence for overlapping, but not identical, protein machineries operating in vegetal RNA localization along early and late pathways in *Xenopus* oocytes. Development 131: 4263–4273. 15294863 10.1242/dev.01283

[32] Zhou Y, King ML (1996) Localization of Xcat-2 RNA, a putative germ plasm component, to the mitochondrial cloud in *Xenopus* stage I oocytes. Development 122: 2947–2953. 8787767 10.1242/dev.122.9.2947

[33] Kloc M, Bilinski S, Chan APY, Etkin LD (2000) The targeting of Xcat2 mRNA to the germinal granules depends on a cis-acting germinal granule localization element within the 3′ UTR. Developmental Biology 217: 221–229. 10625548 10.1006/dbio.1999.9554

[34] Berekelya LA, Mikryukov AA, Luchinskaya NN, Ponomarev MB, Woodland HR, et al.. (2007) The protein encoded by the germ plasm RNA Germes associates with dynein light chains and functions in *Xenopus* germline development. Differentiation E. Pub. 10.1111/j.1432-0436.2006.00160.x 17309602

[35] Hames RS, Hames R, Prosser SL, Euteneuer U, Lopes CAM, et al (2008) Pix1 and Pix2 are novel WD40 microtubule-associated proteins that colocalize with mitochondria in *Xenopus* germ plasm and centrosomes in human cells. Experimental Cell Research 314: 574–589. 18068700 10.1016/j.yexcr.2007.10.019

[36] Wang C, Lehmann R (1991) Nanos is the localized posterior determinant in *Drosophila* . Cell 66: 637–647. 1908748 10.1016/0092-8674(91)90110-k

[37] Lai FF, Singh A, King ML (2012) *Xenopus* Nanos1 is required to prevent endoderm gene expression and apoptosis in primordial germ cells. Development 139: 1476–1486. 22399685 10.1242/dev.079608 PMC3308181

[38] Lai F, Zhou Y, Luo X, Fox J, King ML (2011) Nanos1 functions as a translational repressor in the *Xenopus* germline. Mech Dev 128: 153–163. 21195170 10.1016/j.mod.2010.12.001 PMC3065925

[39] Bontems F, Stein A, Marlow F, Lyautey J, Gupta T, et al (2009) Bucky Ball organizes germ plasm assembly inzebrafish. Current Biology 19: 414–422. 19249209 10.1016/j.cub.2009.01.038

[40] Marlow FL, Mullins MC (2008) Bucky ball functions in Balbiani body assembly and animal-vegetal polarity in the oocyte and follicle cell layer in zebrafish. Developmental Biology 321: 40–50. 18582455 10.1016/j.ydbio.2008.05.557 PMC2606906

[41] Zearfoss NR, Chan AP, Wu CF, Kloc M, Etkin LD (2004) Hermes is a localized factor regulating cleavage of vegetal blastomeres in *Xenopus laevis* . Dev Biol 267: 60–71. 14975717 10.1016/j.ydbio.2003.10.032

[42] Song HW, Cauffman K, Chan AP, Zhou Y, King ML, et al (2007) Hermes RNA-binding protein targets RNAs-encoding proteins involved in meiotic maturation, early cleavage, and germline development. Differentiation 75: 519–528. 17309605 10.1111/j.1432-0436.2006.00155.x

[43] Richardson PT, Hussain K, Woodland HR, Lord JM, Roberts LM (1991) The effects of N-glycosylation on the lectin activity of recombinant ricin B chain. Carbohydr Res 213: 19–25. 1718601 10.1016/s0008-6215(00)90594-9

[44] Messitt TJ, Gagnon JA, Kreiling JA, Pratt CA, Yoon YJ, et al (2008) Multiple kinesin motors coordinate cytoplasmic RNA transport on a subpopulation of microtubules in *Xenopus* oocytes. Dev Cell 15: 426–436. 18771961 10.1016/j.devcel.2008.06.014 PMC2581415

[45] Gerber WV, Yatskievych TA, Antin PB, Correia KM, Conlon RA, et al (1999) The RNA-binding protein gene, hermes, is expressed at high levels in the developing heart. Mechanisms of Development 80: 77–86. 10096065 10.1016/s0925-4773(98)00195-6

[46] Kosaka K, Kawakami K, Sakamoto H, Inoue K (2007) Spatiotemporal localization of germ plasm RNAs during zebrafish oogenesis. Mechanisms of Development 124: 279–289. 17293094 10.1016/j.mod.2007.01.003

[47] Drummond DR, McCrae MA, Colman A (1985) Stability and movement of mRNAs and their encoded proteins in *Xenopus* oocytes. J Cell Biol 100: 1148–1156. 2858488 10.1083/jcb.100.4.1148 PMC2113764

[48] Lewis RA, Kress TL, Cote CA, Gautreau D, Rokop ME, et al (2004) Conserved and clustered RNA recognition sequences are a critical feature of signals directing RNA localization in *Xenopus* oocytes. Mechanisms of Development 121: 101–109. 14706704 10.1016/j.mod.2003.09.009

[49] Kwon S, Abramson T, Munro TP, John CM, Kohrmann M, et al (2002) UUCAC- and Vera-dependent localization of VegT RNA in *Xenopus* oocytes. Current Biology 12: 558–564. 11937024 10.1016/s0960-9822(02)00740-6

[50] Deshler JO, Highett MI, Abramson T, Schnapp BJ (1998) A highly conserved RNA-binding protein for cytoplasmic mRNA localization in vertebrates. Current Biology 8: 489–496. 9560341 10.1016/s0960-9822(98)70200-3

[51] Claussen M, Pieler T (2004) Xvelo1 uses a novel 75-nucleotide signal sequence that drives vegetal localization along the late pathway in *Xenopus* oocytes. Developmental Biology 266: 270–284. 14738876 10.1016/j.ydbio.2003.09.043

[52] MacArthur H, Bubunenko M, Houston DW, King ML (1999) Xcat2 RNA is a translationally sequestered germ plasm component in *Xenopus* . Mechanisms of Development 84: 75–88. 10473122 10.1016/s0925-4773(99)00075-1

[53] MacArthur H, Houston DW, Bubunenko M, Mosquera L, King ML (2000) DEADSouth is a germ plasm specific DEAD-box RNA helicase in *Xenopus* related to eIF4A. Mechanisms of Development 95: 291–295. 10906480 10.1016/s0925-4773(00)00357-9

[54] Heinrich B, Deshler JO (2009) RNA localization to the Balbiani body in *Xenopus* oocytes is regulated by the energy state of the cell and is facilitated by kinesin II. RNA 15: 524–536. 19223445 10.1261/rna.975309 PMC2661827

[55] Glotzer JB, Saffrich R, Glotzer M, Ephrussi A (1997) Cytoplasmic flows localize injected oskar RNA in *Drosophila* oocytes. Current Biology 7: 326–337. 9115398 10.1016/s0960-9822(06)00156-4

[56] Wolke U, Jezuit EA, Priess JR (2007) Actin-dependent cytoplasmic streaming in *C. elegans* oogenesis. Development 134: 2227–2236. 17507392 10.1242/dev.004952

[57] Hachet O, Ephrussi A (2004) Splicing of oskar RNA in the nucleus is coupled to its cytoplasmic localization. 428: 959–963. 10.1038/nature02521 15118729

[58] Gallo CM, Wang JT, Motegi F, Seydoux G (2010) Cytoplasmic partitioning of P granule components is not required to specify the germline in *C. elegans* . Science 330: 1685–1689. 21127218 10.1126/science.1193697 PMC3072820

[59] Brangwynne CP, Eckmann CR, Courson DS, Rybarska A, Hoege C, et al (2009) Germline P granules are liquid droplets that localize by controlled dissolution/condensation. Science 324: 1729–1732. 19460965 10.1126/science.1172046

[60] Zimyanin VL, Belaya K, Pecreaux J, Gilchrist MJ, Clark A, et al (2008) In vivo Imaging of oskar mRNA transport reveals the mechanism of posterior localization. Cell 134: 843–853. 18775316 10.1016/j.cell.2008.06.053 PMC2585615

[61] Hird SN, Paulsen JE, Strome S (1996) Segregation of germ granules in living *Caenorhabditis elegans* embryos: Cell-type-specific mechanisms for cytoplasmic localisation. Development 122: 1303–1312. 8620857 10.1242/dev.122.4.1303

[62] Guedes S, Priess JR (1997) The *C. elegans* MEX-1 protein is present in germline blastomeres and is a P granule component. Development 124: 731–739. 9043088 10.1242/dev.124.3.731

[63] Zhang Y, Yan L, Zhou Z, Yang P, Tian E, et al (2009) SEPA-1 mediates the specific recognition and degradation of P granule components by autophagy in C. elegans. Cell 136: 308–321. 19167332 10.1016/j.cell.2008.12.022

[64] Mosquera L, Forristall C, Zhou Y, King ML (1993) A messenger-RNA localized to the vegetal cortex of *Xenopus* oocytes encodes a protein with a Nanos-like zinc finger domain. Development 117: 377–386. 8223259 10.1242/dev.117.1.377

[65] Dale L, Matthews G, Tabe L, Colman A (1989) Developmental expression of the protein product of Vg1, a localized maternal messenger RNA in the frog *Xenopus laevis* . Embo Journal 8: 1057–1065. 2519512 10.1002/j.1460-2075.1989.tb03473.x PMC400914

